# Adhesives Inspired by Marine Organisms: Multifunctional Design and Application for Complex Wet/Underwater Environments

**DOI:** 10.3390/polym18202585

**Published:** 2026-10-20

**Authors:** Haonan Chen, Ningchuan Yang, Yanlin Chen, Yijun Liu, Jiuyin Pang, Xiaoyi Niu

**Affiliations:** 1 School of Materials Science and Engineering, Beihua University, Jilin 132013, China; 2 Jilin Provincial Key Laboratory of Wood Materials and Science, Jilin 132013, China

**Keywords:** adhesive, bionics, wet adhesion, polymer

## Abstract

Achieving strong adhesion in humid or underwater environments is a long-term challenge in materials science. Water molecules form a hydration layer on the substrate surface, blocking effective contact between the adhesive and the interface, leading to the failure of traditional adhesives. The wet adhesion strength of viscoelastic materials is due to the synergistic effect of interfacial chemical bonds and multiscale energy dissipation, but water environment can seriously interfere with this mechanism. In nature, mussels, barnacles, and sandcastle worms have evolved unique wet adhesion strategies: mussels achieve interfacial bonding through metal coordination and oxidative cross-linking of catechol groups; barnacle adopts a completely different “phase separation solidification” pathway, in which its gelatinous protein complex undergoes liquid–liquid phase separation to form a dilution-resistant coacervate that spreads with extremely low surface tension and displaces the interfacial hydration layer. Sandcastle worms use polyelectrolyte composite coagulation to bond mineral particles. Inspired by this, the synthetic pathways of biomimetic wet adhesives include the synthesis of peptides, modified polymers, and polydopamine systems, which have shown promising applications in wearable electronic devices, marine engineering restoration, and biomedical fields. This article systematically reviews the progress of biomimetic materials, providing reference for the rational design of high-performance adhesive materials.

## 1. Introduction

Achieving strong adhesion in underwater or humid environments remains one of the long-standing core challenges in the field of materials science. The bonding mechanism of traditional adhesives relies heavily on solvent evaporation and surface drying conditions [1,2,3]. However, in wet environments, the surfaces of the substrates are often covered by a dense hydrated layer. This thin film of water molecules acts as an invisible barrier, preventing direct molecular contact between the adhesive and the substrate, which significantly weakens interfacial bonding [4,5,6,7,8]. Even more challenging is the fact that the continuous presence of surrounding water dilutes the adhesive’s active ingredients, leading to a decrease in its cohesive strength [9]. For highly hydrophilic materials, water penetration can also trigger swelling and plasticization effects, further compromising the mechanical properties of the cured adhesive. Data show that the adhesion strength of conventional adhesives in wet conditions is often less than 10 kPa—lower even than that of ordinary adhesive tape in dry environments—which falls far short of the practical requirements in engineering or medical applications [10,11,12,13]. Furthermore, wet-state applications involve substrates with diverse physical structures and chemical properties. From dense, non-porous inorganic materials to biological tissues with water content as high as 70%, and from porous polymer gels to structures covered by a lipid layer, the adhesion requirements for different substrates vary greatly, further complicating the development of universal, high-performance wet-state adhesives [14,15,16,17,18]. Although synthetic materials have repeatedly faced setbacks in the field of wet-state adhesion, nature has long provided an ingenious solution. Over the course of a long evolutionary history, various marine organisms inhabiting intertidal and shallow marine environments have evolved the extraordinary ability to adhere firmly under wet or even fully submerged conditions [19]. Among them, the three most representative groups of organisms have each developed unique wet-adhesion strategies, providing an irreplaceable source of inspiration for the design of artificial adhesives [20,21,22].

Mussels anchor themselves to wet rocks, ship hulls, and even polymer surfaces by secreting a byssus disc composed of various byssus filament proteins. Even under repeated wave impact, these adhesive structures remain stable, thanks primarily to a specific amino acid: 3,4-dihydroxy-L-phenylalanine (DOPA). The catechol groups in its side chains form strong hydrogen bonds with the hydrated substrate surface, while coordination with metal ions and oxidative cross-linking reactions enable synergistic interfacial bonding and cohesive curing through multiple mechanisms [23,24,25,26]. Barnacles, on the other hand, employ a completely different strategy: they secrete a protein complex known as “barnacle glue,” which undergoes liquid–liquid phase separation after secretion, forming a coagulant that repels seawater dilution. By spreading and penetrating the substrate surface, it eventually cross-links and cures into a dense adhesive patch, thereby achieving permanent and robust attachment [27]. Sandcastle worms, on the other hand, utilize polyelectrolyte complexation, mixing oppositely charged proteins and polysaccharides to form a fluid-like, water-immiscible adhesive gel. They use this to bond sand grains and shell fragments while wet, constructing the tubular protective shells in which they reside [28,29,30,31].

Inspired by the marine organisms mentioned above, researchers have conducted extensive and systematic work on biomimetic wet adhesives over the past two decades. This work has generally followed two complementary approaches [32,33,34,35]. The first is chemical biomimicry, which involves simulating the molecular interactions of key functional groups in natural adhesion proteins; the second is physical/structural biomimicry, which involves mimicking the micro and nanostructures and phase-transition behaviors of biological adhesive organs. In the field of chemical biomimicry, catechol chemistry undoubtedly occupies the most central position [36,37,38]. Researchers have incorporated dopamine or molecules containing catechol groups into various polymer frameworks, synthesizing a large number of mussel-inspired polymer adhesives with different molecular structures and functions [39,40,41]. These materials not only exhibit significantly enhanced interfacial adhesion in wet environments but also possess both good cohesive strength and self-healing properties, as their catechol groups can participate in reversible non-covalent interactions and irreversible covalent cross-linking [42].

In recent years, there have been several noteworthy new trends in the field of biomimetic wet adhesives. One approach is data-driven design: Liao et al. [43] analyzed the sequences of 24,707 adhesins from different sources, revealing a common pattern of efficient adhesin amino acid composition. By using machine learning to accelerate the iterative optimization of materials, they finally developed an underwater super viscous hydrogel, whose adhesion strength reached the range of MPa. Tan et al. [44] developed an intelligent responsive adhesive that enables the adhesive to switch between bonding and separation based on external stimuli such as temperature, pH, and electric field. This is particularly important for applications such as temporary tissue fixation and repeated attachment of wearable sensors in minimally invasive surgery. The third is the extension of non-DOPA dependent systems. Wang et al. [45] discovered a scallop foot line protein composed of epidermal growth factor domains, which can rapidly self-assemble into extracellular matrix-like coatings under Ca^+^. This coating does not rely on traditional DOPA chemistry, but exhibits equally excellent wet adhesion performance; Compared with existing commercial products, its antioxidant and antibacterial activities show superior wound healing effects in the treatment of chronic diabetes wounds, opening up a new molecular path for the design of wet adhesive materials. In addition, Wang et al. [46] conducted a systematic study on ionic gel. Through the engineering design of dynamic complementary cross-linking networks, the catechol free adhesive can maintain a stable adhesive strength of over 3.3 MPa in environments ranging from freshwater to high salinity saltwater (up to 200 g·kg^−1^), providing a highly promising candidate material for marine engineering applications.

In summary, with an increasingly deep understanding of the molecular mechanisms underlying natural adhesion systems, the next generation of biomimetic wet-state adhesives is expected to achieve a qualitative leap in performance, functionality, and accessibility. In the following sections, this paper will systematically review the wet-state adhesion mechanisms of major marine organisms—such as mussels, barnacles, and sandcastle worms—and their biomimetic design strategies; categorize and evaluate the latest research findings and key performance indicators of various biomimetic wet-state adhesives; and thoroughly explore cutting-edge application directions and associated challenges, with the aim of providing a comprehensive reference for future research in this field [47,48,49,50,51,52].

## 2. Adhesion Mechanism

Unlike terrestrial adhesion that relies on dry interfaces and solvent evaporation, wet adhesion faces challenges due to the adsorption of water molecules at the solid–liquid interface. When the surface of the material is immersed in water, water molecules instantly form a hydration layer on its surface that is several nanometers to tens of nanometers thick [46,53,54]. This layer of structure is tightly bound to the interface, becoming a barrier that hinders the contact between the adhesive and the substrate. Even if the adhesive can break through the contact of the hydration layer, the subsequent curing process must still resist the dilution effect of surrounding water, prevent the hydrolysis or premature dissolution of active groups, and maintain the cohesive strength of the adhesive layer itself. Therefore, an effective wet adhesion system must achieve synergy and balance in the three key links of interface drainage, colloid protection, and heterogeneous solidification [55,56,57,58]. Based on the above challenges, marine invertebrates have evolved two adhesion pathways: one is the chemical adhesion strategy represented by mussels, which relies on the formation of strong non-covalent and covalent interactions between interfacial active molecules and the hydration layer, directly “competing” for local surface binding sites. Another type is the phase transition adhesion strategy represented by barnacles and sandcastle worms. Encapsulate the active adhesive component in a condensed phase that is immiscible with water. Firstly, the water layer is spread out through physical means, and then cured in situ to form a permanent bond. At the same time, some vertebrates such as tree frogs, octopuses, and throat fish have also evolved physical adhesion modes based on micro nano structures, providing a reference for reversible and on-demand adhesion in wet conditions [59,60,61,62,63].

### 2.1. Mussels

**Figure 1:**
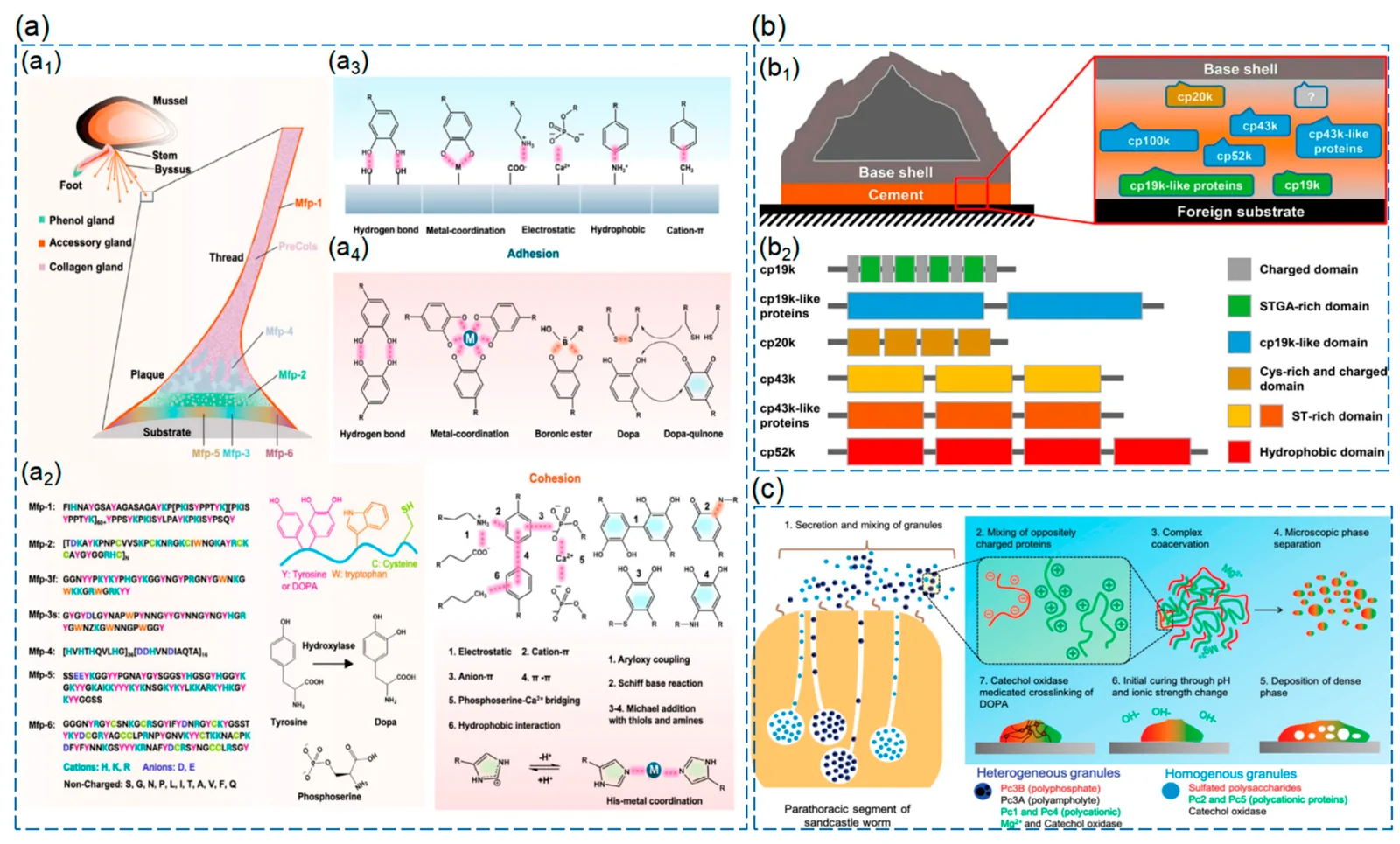
Adhesion mechanisms of marine organisms: mussels [75], barnacles [76], and sandcastle worms [77]. (**a_1_**) Anatomy of a mussel and its byssus, showing the foot, stem, thread, plaque, adhesive plaque, and substrate. The distribution of mussel foot proteins (Mfp−1 to Mfp−6) is indicated: Mfp−3 and Mfp−5 are mainly responsible for interfacial adhesion; Mfp−1 acts as an outer protective coating; Mfp−2 and Mfp−4 contribute to crosslinking and mechanical buffering inside the plaque. (**a_2_**) Primary sequences of six Mfps, with DOPA (Y, 3,4−dihydroxy-L−phenylalanine), tryptophan (W), and cysteine (C) highlighted. (**a_3_**) Interfacial adhesion mechanisms of mussel proteins: hydrogen bonding, metal coordination, electrostatic interaction, hydrophobic interaction, and cation−π interaction. These interactions displace the interfacial hydration layer and enable wet adhesion. (**a_4_**) Cohesive mechanisms within the mussel plaque: hydrogen bonding, metal coordination, borate ester formation, DOPA−DOPA coupling, disulfide formation, Michael addition, Schiff base reaction, and His−metal coordination. (**b_1_**) Barnacle adhesion to a foreign substrate. The barnacle secretes cement through its base shell; interface proteins (cp19k, cp20k, cp43k/cp43k−like) are enriched near the substrate, while bulk proteins (cp52k, cp100k) form the main cement matrix. Cp19k-like proteins may distribute in both interface and bulk regions. (**b_2_**) Domain organization of representative barnacle cement proteins, including charged domains, STGA−rich domains, cp19k−like domains, Cys−rich/charged domains, BT−rich domains, and hydrophobic domains. These domains underlie self−assembly, amyloid-fiber formation, and enzymatic crosslinking. (**c**) Underwater adhesion mechanism of sandcastle worms. Heterogeneous granules contain Pc3A, sulfated polysaccharides, and polyphosphates; homogeneous granules contain cationic proteins Pc1, Pc2, Pc4, and Pc5. After secretion and mixing, oppositely charged proteins/polysaccharides undergo complex coacervation and microscale phase separation, forming a dense adhesive phase. This dense phase spreads on the substrate, dehydrates upon pH/ionic-strength changes, and finally solidifies through catechol oxidase- and Mg^2+^−mediated DOPA oxidation, covalent crosslinking, and metal coordination. Arrows indicate the sequential adhesion process.

Marine mussels can secrete multiple foot filaments (2–6 cm in length and 100–200 μm in diameter) from their foot organs within a few minutes, which attach to the marine substrate. Foot silk is mainly composed of various mussel foot proteins (Mfps), which are distributed in different regions according to functional differences and consist of three parts: stem, silk, and plaque (Figure 1a_1_). The stem is connected to the foot of the mussel, and the molecular components of the foot silk thread are generated in the foot and stored in specialized glands. The slender middle section of the foot contains two regions: the proximal end has elastic characteristics and can withstand periodic and strong mechanical loads caused by waves; The distal end is harder and connected to adhesive. Mussels rely on their foot silk discs to anchor to a moist substrate, and various Mfps are densely distributed at the interface between the foot silk discs and the substrate. Among them, Mfp-3 and Mfp-5 mainly undertake interface adhesion; Mfp-1, as an external protective coating, can prevent wear and UV damage; Mfp-2 and Mfp-4 are responsible for cross-linking and mechanical buffering inside the footplate (Figure 1a_2_). Among these protein families, the unusually abundant post-translationally modified amino acid 3,4-dihydroxy-L-phenylalanine (DOPA), generated by hydroxylation of tyrosine residues, is the core functional unit of the mussel adhesion system. The catechol groups on the DOPA side chain have both hydrogen bond donor and acceptor interactions (Figure 1a_3_), and possess hydrophobic planes due to the benzene ring, which makes them exhibit significant multifunctionality in interfacial chemical adsorption. For moist inorganic oxide surfaces such as titanium oxide, mica, and glass, catechol groups can form reversible coordination bonds with unsaturated metal atoms on the surface; its binding energy is much higher than the hydrogen bonds between water molecules and the same surface site, thus it can effectively displace the hydration layer [64,65,66,67,68,69]. On the surface of organic substrates, catechol benzene rings can adhere through π–π stacking and hydrophobic interactions. What is even more unique is that catechol groups can be oxidized to highly active quinone structures under pH or oxidative stimulation, and then undergo Michael addition or Schiff base reactions with nucleophilic groups such as amino and thiol groups on the substrate surface. Ultimately, an irreversible covalent bond is formed, transforming the initial reversible attachment into permanent anchoring. This temporal adhesion program of “reversible recognition followed by irreversible locking” is the key to achieving long-term high-strength adhesion of mussels to wet substrates [70,71,72,73,74].

However, there is an inherent “oxidation conflict” between the interfacial adhesion and cohesive crosslinking of DOPA (Figure 1a_4_). The reduced state of catechol is conducive to the formation of interfacial hydrogen bonds and coordination bonds, but once oxidized to orthoquinone, its hydrogen bonding ability is greatly weakened, and it tends to participate in covalent crosslinking between protein molecules, enhancing the cohesion of the adhesive layer. Excessive oxidation may harden the adhesive layer, but at the cost of sacrificing interfacial adhesion. Mussels cleverly resolve this contradiction through various regulatory measures. The microenvironment at the interface of Mfp-3 and Mfp-5 maintains weak acidity, effectively inhibiting the spontaneous oxidation of catechol [78]. Mfp-6 contains abundant cysteine residues, which can provide thiol electron donors to reduce oxidized quinones back to catechol, thereby maintaining the interface DOPA in a reducing active state. Meanwhile, in the body of the foot silk disc, DOPA of proteins such as mfp-2 can be fully oxidized and cross-linked under alkaline seawater and enzymatic conditions, forming a solidified internal network [79,80,81]. In addition to the core contribution of DOPA, the cation–π interaction between the abundant cationic amino acids (lysine, arginine) in mussel protein and catechol groups has been confirmed to be an important molecular mechanism for assisting interface drainage. Surface force measurement studies have shown that when mfp molecules approach the negatively charged mica surface, cationic residues first penetrate the hydration layer through electrostatic attraction. Subsequently, the catechol benzene ring interacts synergistically with the same surface, squeezing out residual water molecules and establishing short-range adhesive contact. This cation–π synergistic mode has inspired the design of a large number of synthetic biomimetic adhesives. Introducing both quaternary ammonium/amino cationic groups and catechol groups onto the polymer chain can significantly enhance the wet adhesion capacity of the material [82].

### 2.2. Barnacle

Unlike the interface chemical adsorption of mussels, barnacles adopt a completely different strategy of “phase separation before solidification”. The barnacles secrete a protein complex called barnacle cement proteins in their attachment plaques at the base. Morphological and biochemical studies have revealed that barnacle cement proteins exist in the form of concentrated liquid aggregates at the beginning of secretion. This is a dense fluid phase rich in protein and highly dehydrated formed through liquid–liquid phase separation, suspended in seawater but immiscible with it [83,84,85,86]. The surface tension of this condensed phase is extremely low, and it can wet and spread on various substrates in a wet state, directly replacing the hydration layer at the interface without being diluted by seawater. Barnacle cement protein does not contain DOPA, and its interfacial adhesion mainly relies on hydrophobic interactions, amide group hydrogen bonding networks, and electrostatic attraction between cationic residues and negatively charged groups on the substrate surface. After the coalescence is spread, enzymatic cross-linking occurs within the protein complex. Lysyl oxidase catalyzes the formation of aldehyde derivatives from lysine residues, which then form Schiff base cross-linking with adjacent protein lysines [87]. Transglutaminase catalyzes the formation of heteropeptide bonds between glutamine and lysine, accompanied by self-assembly and stacking of hydrophobic protein domains, ultimately forming an amyloid fiber network rich in β-folds. This fiber structure endows barnacles with excellent mechanical strength and hydrolysis resistance, allowing them to persist on ship hulls and rock surfaces for life. The condensation of barnacles naturally avoids seawater dilution and premature dissolution of active groups through phase separation. Its design concept provides important inspiration for the development of biomimetic adhesives that are wet spreadable, erosion resistant, and long-term stable after curing [80,88,89,90].

The key to achieving strong and permanent underwater adhesion of barnacles in marine environments lies in the secretory protein complex composed of various cement proteins (CPs). Figure 1b shows the hierarchical assembly model of barnacle cement protein and the spatial distribution and functional division of its various protein components [76]. From the macroscopic cross-section (marked on the left and bottom scales in Figure 1b_1_), the barnacles secrete adhesive through their calcium substrate shell to the interface between the substrate shell and the external substrate, and achieve permanent adhesion after curing. Barnacle glue is a viscous insoluble protein complex with a protein content of approximately 90% or more. At present, various types of gelatinous proteins have been identified, mainly including cp19k, cp20k, cp43k, cp52k, cp68k, and cp100k. According to their functional localization, these proteins can be divided into two categories: interface proteins and ontology proteins. Figure 1b_2_ shows the molecular structural characteristics and spatial distribution of each collagen protein. At the interface layer (near the foreign substrate), cp19k, cp20k, and cp43k/cp43k-like proteins are mainly enriched here, responsible for the interface adhesion function directly binding to the substrate. Among them, cp19k exhibits typical block copolymer-like sequence characteristics, with its primary structure composed of two alternating segments: one rich in Ser, Thr, Gly, and Ala, and the other dominated by hydrophobic and charged amino acids. Cp20k contains abundant Cys residues, and its primary structure exhibits a repetitive fragment arrangement. Research has shown that cp20k plays an important role in interfacial adhesion, and its disulfide bond is crucial for protein multi-level self-assembly and underwater affinity. Cp43k and its homologous protein (cp43k-like) are also localized in the interface region, with cp43k-like being the most expressed interface protein [91,92,93,94].

In the bulk adhesive layer (cement region), cp52k and cp100k are the main bulk proteins. The sequence of cp52k consists of four large repeating units and contains a hydrophobic domain, while cp100k exhibits a high degree of homogeneity in amino acid composition, isoelectric point, and hydrophobicity, suggesting that it plays an irreplaceable role as a core component of barnacle cement protein [95,96,97,98]. These host proteins self-assemble to form amyloid-like fibers, providing cohesive strength for the adhesive layer. It is worth noting that the cp19k-like protein is labeled in the figure as being distributed simultaneously in both the interface and the host region, suggesting that it may have dual functions of interface adhesion and host aggregation. The genes cp19k, cp20k, cp43k, and cp52k have undergone significant repetitive events during evolution, and the number of internal repetitive motifs varies among different species, reflecting the plasticity and evolutionary adaptability of these protein functions [82,90,99,100,101,102].

### 2.3. Sandcastle Worm

The underwater adhesive used by sandcastle worms to collect sand particles and shell debris to construct their habitat represents another classic wet adhesion model of condensed matter in the biological world. This adhesive is formed by the polyelectrolyte complex condensation of positively charged proteins (rich in glycine, tyrosine, and lysine) and negatively charged proteins (rich in phosphorylated serine) in secretory glands at pH and counterion concentration. After the formation of aggregates, they become viscoelastic fluids. After injection into the gaps between sand particles, they undergo ion crosslinking through the coordination of calcium and magnesium ions in seawater with phosphorylated serine residues [103,104,105]. At the same time, tyrosine residues are oxidized to dopaquinone-like structures under the catalysis of polyphenol oxidase and participate in covalent solidification, ultimately forming mechanically stable tubular structures. The phosphorylation of serine in this system is similar to DOPA in mussels [106]. Phosphate groups also have strong metal coordination and hydrogen bonding abilities, which can achieve firm adsorption on the surfaces of calcium or siliceous minerals. More importantly, the polyelectrolyte composite coagulation itself provides natural waterproof protection, and the water content in the coagulation body separated from the water phase is extremely low, ensuring the high efficiency of crosslinking reaction and high cohesion of the cured network. This mechanism directly gave rise to the design trend of synthetic polyelectrolyte aggregate adhesives and has been further promoted to multi biopolymer systems such as protein polysaccharides [107].

As shown in Figure 1c, the underwater adhesion process of the sandcastle worm begins with the coordinated release of two types of secretory particles within its thoracic segment [77]. Homogeneous particles carry cationic proteins (Pc1, Pc2, Pc4, Pc5), while heterogeneous particles are rich in polyelectrolyte proteins (Pc3A), sulfated polysaccharides, and polyphosphates (Pc3B). Once the two are mixed (Step 2), proteins and polysaccharides with opposite charges quickly undergo complex aggregation (Step 3), forming a dense liquid aggregate that is insoluble in seawater but has fluidity. The condensed material undergoes further microscale phase separation (Step 4), forming a dense phase rich in adhesive proteins, laying the foundation for subsequent spreading and deposition on the substrate surface. The dense phase of the condensed material is subsequently deposited onto the substrate surface (Step 5). Upon contact with seawater, the increase in environmental pH and changes in ionic strength (Step 6) promote the initial dehydration and solidification of the condensed material, increasing its cohesive strength. Finally, under the co mediation of catechol oxidase and Mg^2+^ (Step 7), DOPA residues in the protein are oxidized to benzoquinone, triggering intermolecular covalent crosslinking and forming metal coordination bonds with the substrate surface, completing the final curing of the adhesive [43,108,109].

### 2.4. Summary

The comparison of adhesion mechanisms between mussels, barnacles, and sandcastle worms is shown in Table 1, representing three distinct wet adhesion strategies. Mytilus edulis relies on the catechol chemistry of DOPA in its foot silk protein to achieve a “reversible recognition followed by irreversible locking” temporal adhesion through metal coordination and oxidative cross-linking. Its interface bonding combines hydrogen bonding, π–π stacking, and cation–π synergistic effects. However, there is an inherent conflict between the reduced state (conducive to adhesion) and the oxidized state (conducive to cohesion) of DOPA, which requires the regulation of microenvironmental pH and Mfp-6 thiol reduction to maintain balance [110].

Barnacle, on the other hand, adopts a completely different “phase separation solidification” pathway, in which its gelatinous protein complex is separated into resistant to dilution aggregates through liquid–liquid phase separation, and the interfacial water layer is expelled with extremely low surface tension. Subsequently, a permanent high-strength adhesive layer was constructed by catalyzing cross-linking with lysyl oxidase and transglutaminase, supplemented by self-assembly of β-folded amyloid fibers, without relying on DOPA throughout the entire process. The adhesion of sandcastle worms is based on polyelectrolyte composite coagulation, which is triggered by seawater to form fluid-like aggregates of positively charged and negatively charged proteins. Rapid solidification is achieved through Mg^2+^/Ca^2+^ coordination and tyrosine oxidation cross-linking. Its phosphorylated serine has both negative charge supply and mineral adsorption functions [111,112,113,114].

## 3. Interface Adhesion Characterization Methods

### 3.1. Macro Mechanical Performance Testing

**Figure 2:**
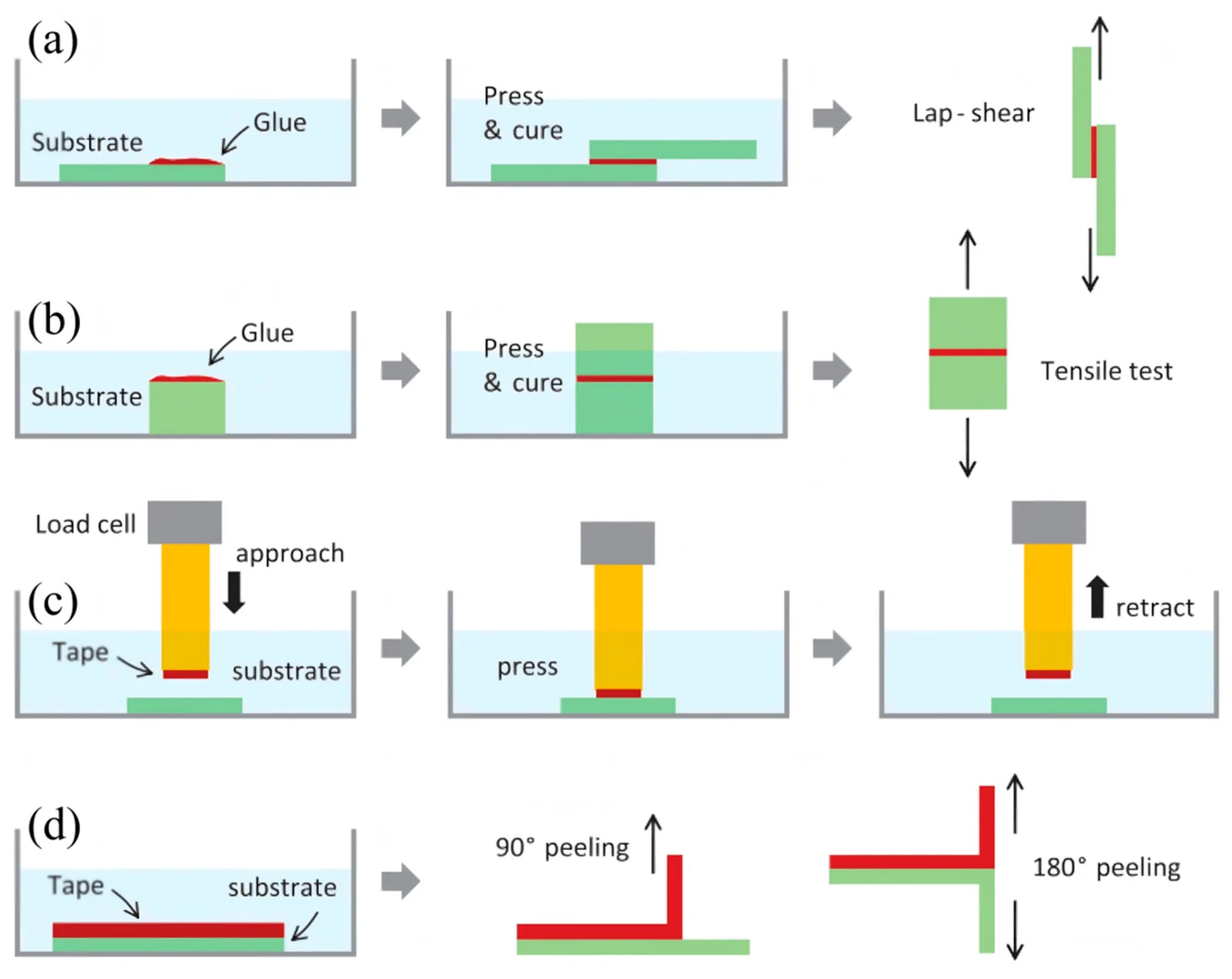
Schematic diagram of common macroscopic testing methods for underwater bonding [123]. Blue regions indicate water. Arrows indicate loading directions. (**a**) Lap-shear test. Adhesive is applied to a substrate, pressed and cured underwater, and then loaded in shear parallel to the bond line. This test evaluates lap-shear strength and interfacial load-bearing capacity. (**b**) Tensile test. Adhesive is placed between two substrates, pressed and cured, and then pulled perpendicular to the bond line. This measures tensile adhesion strength and cohesive failure behavior. (**c**) Probe tack test. A load-cell-mounted probe with tape approaches the wet substrate, presses with a controlled preload, and retracts. (**d**) Peel test, including 90° and 180° configurations. A flexible tape or adhesive is attached to a wet substrate and peeled at a specific angle. The recorded peel force evaluates peel resistance and interfacial toughness.

In recent years, research on biomimetic underwater adhesives has extended from material design to testing methodology. Figure 2 summarizes the evaluation of underwater adhesion into lap shear, tensile, adhesive, and peel. Lap shear and tensile testing are the fundamental methods for evaluating load-bearing underwater adhesives. This method is mainly used to evaluate the shear resistance of adhesives in the direction parallel to the bonding interface [115]. As shown in Figure 2a, first apply the adhesive onto the substrate, then cover another substrate and press cure to form a lap structure. Subsequently, the testing instrument applies opposite tensile forces to the two substrates in a direction parallel to the bonding surface, causing them to slide relative to each other, in order to measure the strength of the adhesive against shear stress in a wet environment. Stretching is used to measure the tensile strength of adhesives in the direction perpendicular to the bonding interface. As shown in Figure 2b, apply glue between two substrates, and after pressing and curing, form a sandwich-like sandwich structure. During testing, the instrument applies tension along the direction perpendicular to the bonding surface (upper and lower ends), directly stretching the bonding interface until the joint fractures, in order to evaluate the adhesive’s ability to resist positive tensile (normal) stress in the wet state. In recent years, researchers have emphasized the use of underwater methods for coating, pressing, and curing to simulate actual construction [116,117,118]. The sandcastle worm-like adhesive developed by Wang et al. [119] achieved a lap shear strength of 498.42 kPa after curing underwater for 72 h, and remained at 451.95 kPa after 50 days of immersion. Fan et al. [120] designed super viscous hydrogels by data-driven method, and systematically studied the relationship between tensile behavior and network topology. The amphibious adhesive imitating barnacles and articular cartilage achieved an underwater lap shear strength of 4.2 MPa.

Researchers revealed the synergistic mechanism of hydration layer failure and nanosheet diffusion barrier through multi-scale analysis. The lap shear strength of P3/SiW condensate on titanium substrate reached 101.1 kPa, and the force displacement curve showed ductile fracture characteristics. The researchers confirmed the existence of interface coordination bonds through XPS analysis. The comparison of shear strength and testing conditions of representative biomimetic wet adhesives is shown in Table 2.

The probe tack test method is shown in Figure 2c. This method quantifies the adhesion force of the wet interface through the process of contact press separation. As shown in the lower left corner of the figure, the testing system includes a probe connected to the load sensor. During testing, the probe first approaches and presses onto the substrate with adhesive tape, and then retracts upwards. By recording the changes in force and displacement during the retraction process, the interface adhesion force between the probe and the wet substrate can be accurately measured. Fan et al. [121] evaluated the underwater adhesion performance of biomimetic sandcastle worm copolymers (BA co AA) with different AA contents using a viscosity testing system and found that p (BA_28_-AA_1_._0_) has the best comprehensive performance, with an underwater bonding strength of 56.9 kPa and a debonding work of 45.1 J/m^2^. Rao et al. [122] studied the adhesion behavior of PA hydrogels with different surface patterns to glass and soft PA gel substrates under water by using viscosity tests, and found that samples with hexagonal patterns showed higher debonding force and deformation on soft substrates, revealing the coupling effect of interface bridging strength and substrate deformation ability. Another advantage of the tape press back test is that it can evaluate reversibility through cyclic loading—the sample can recover about 90% of its adhesive strength and debonding work within 1 min. The researchers also used this technique to investigate the effects of preload and contact time on adhesion strength, and found that the adhesion strength increased from 50 kPa to 238 kPa when the preload was increased from 10 N to 100 N.

As shown in Figure 2d, the peel test includes two configurations: 90° and 180° [123]. This method is used to evaluate the peel resistance of flexible tape under wet conditions. As shown on the bottom right side of the figure, during testing, the tape is attached to the surface of the substrate, and then the tape is torn off from the substrate at a specific angle. The testing instrument will record the force during the peeling process to analyze the peeling strength of the tape at the wet interface. The researchers found that when a strengthening anchor is formed between the hydrogel and the substrate, the interfacial toughness on the substrate reaches 230 J/m^2^, and the lap shear strength reaches 56 kPa; On rough surfaces such as paper and textiles, the interfacial toughness exceeds 400 J/m^2^ and the shear strength exceeds 190 kPa [124,125,126]. The researchers also simulated marine environmental conditions and subjected the samples to a static water pressure of 4 bar for 1 h before conducting a 180° peel test to verify the stability of adhesion performance under pressure conditions [127]. The polyampholyte hydrogel and glass fiber fabric composite prepared by King et al. [128] achieved effective interfacial toughness of up to 250 kJ/m^2^ through fiber pull-out mechanism. In terms of data processing, 180° stripping requires consideration of double platform force contribution, while 90° stripping is considered by some researchers to provide more “pure” measurement results due to its lower energy dissipation contribution. Overall, current testing methods are moving from a single intensity indicator to multi indicator, multi-mode, and multi environment evaluation. The researchers’ work indicates that future research progress will focus on establishing underwater adhesion testing standards, developing in situ real-time visualization testing platforms, and integrating shear, tensile, adhesion, and peel data into interface constitutive models, thereby promoting the practical application of biomimetic underwater adhesives.

### 3.2. Micro Nano Scale Testing

#### 3.2.1. Atomic Force Microscopy Colloidal Probe Force Spectroscopy Technology

**Figure 3:**
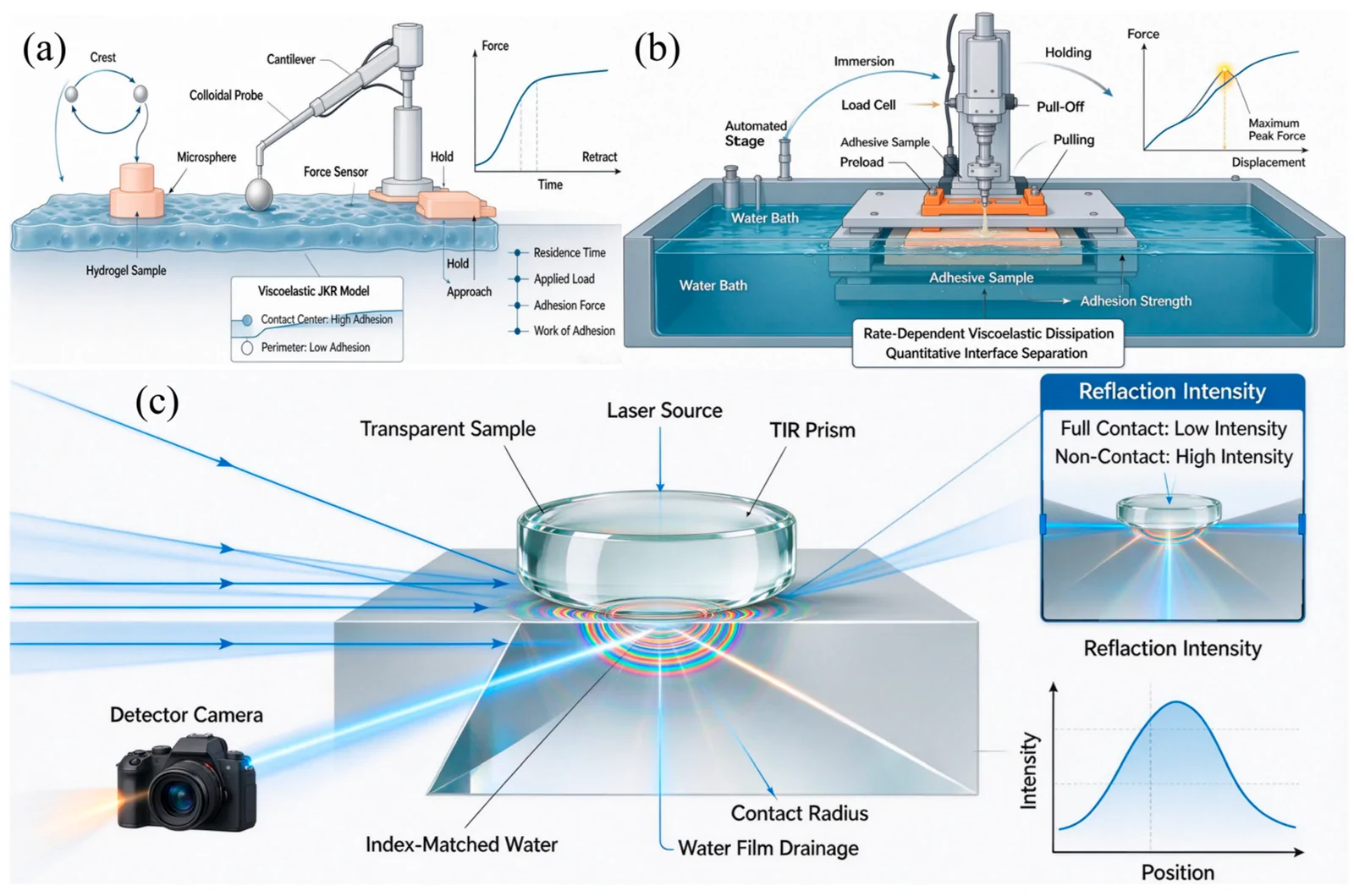
Micro/nanoscale testing devices for wet adhesion. (**a**) Atomic force microscopy (AFM)-based colloidal-probe force spectroscopy for micro/nanoscale wet adhesion measurement. A microsphere probe attached to a cantilever approaches, contacts, dwells on, and retracts from a hydrogel or soft adhesive in a liquid environment. The force–time/displacement curve provides adhesion force, work of adhesion, and viscoelastic relaxation time. The inset shows a viscoelastic Johnson–Kendall–Roberts (JKR)-type contact model, in which adhesion energy is non-uniformly distributed over the contact area: strongest at the center and gradually disappearing at the periphery. (**b**) Wet peel/pull-out fixture for macroscopic interface adhesion characterization. An adhesive sample is immersed in a water bath and mounted on an automated stage. A load cell pulls the sample/probe, and the force–displacement curve gives maximum force and adhesion strength. The system can evaluate rate-dependent viscoelastic dissipation and quantitative interfacial separation. (**c**) In situ wet-adhesion observation system based on optical interference and suppressed total internal reflection imaging. Laser light passes through a total internal reflection (TIR) prism; a transparent sample contacts the prism through index-matched water. A detector camera records reflected intensity. Full contact gives low reflection intensity, while non-contact areas give high intensity. The technique tracks contact radius and water-film drainage in real time. The inset shows reflection intensity versus position, and the light path is indicated by arrows.

In the study of wet adhesion mechanics at the microscale, the force spectrum technique combining atomic force microscopy with colloidal probes is one of the most direct and sensitive characterization methods [129]. Atomic force microscopy colloidal probe force spectroscopy is a highly sensitive characterization method for micro nano wet state adhesion mechanics. The testing device diagram is shown in Figure 3a. Microsphere probes come into contact with hydrogels or soft adhesive samples according to force control procedures in the liquid environment. Real time collection of force response between the probe and the sample during the entire process of probe approach, residence, and retraction, obtaining quantitative parameters such as adhesion force, adhesion work, and viscoelastic relaxation time. Combined with the viscoelastic extended contact model, this method can reveal the nano localized interface adhesion regulated by water molecules, achieving high spatial resolution testing of single-cell adhesion [130]. The latest research shows that the viscoelastic expansion of the classic Johnson Kendall Roberts contact mechanics model can accurately describe the dynamic adhesion behavior of hydrogels under different dwell times and applied loads. The model assumes that the distribution of adhesion energy on the contact area is uneven, with the strongest at the contact center and gradually disappearing at the periphery. The evolution of viscoelastic and mechanical parameters with holding time and load derived from this is highly consistent with experimental data. AFM based force spectrum technology is suitable for revealing how water molecules affect adhesion locally at interfaces by changing the spreading conformation and permeation rate of polymer chains due to its nanoscale force sensitivity and micrometer level spatial resolution [131,132,133].

In recent years, researchers have made significant progress in probe functionalization and measurement strategies. Beaussart et al. [134] proposed a non-destructive single bacterial force spectrum scheme, which uses biomimetic polydopamine wet adhesives to adhere live probiotics (*Lactobacillus plantarum*) to colloidal probes, achieving quantitative measurement of adhesion force between individual bacteria and lectin monolayers or hydrophobic monolayers, providing a universal platform for the study of molecular mechanisms of bacterial adhesion. At the quantitative analysis level, Zhang et al. [135] used colloidal probe AFM to quantitatively measure the interaction force and adhesion energy between polydopamine coatings and substrates with different wettability. They found that hydrophobic surfaces exhibited stronger PDA adhesion due to the presence of a “hydrophobic depletion layer”, which could be effectively replaced by ethanol to enhance interfacial contact. More noteworthy is the establishment of a dual scale force spectrum method: researchers combined the mesoscopic soft colloid probe (SCP) force spectrum with the nanoscale AFM single chain force spectrum, using the polylysine polyglutamic acid complex as a model system, to reveal how the solid–liquid phase transition driven by hydrogen bond network disruption transforms nanoscale brittle chain fracture into high ductility viscous stretching, thereby significantly enhancing macroscopic adhesion energy. This method elucidates the key regulatory role of interfacial fluidity on wet adhesion, providing important principles for the design of underwater adhesives. Lionel et al. [136] developed a method for characterizing single-cell adhesion properties by using protein functionalized AFM probes. The quantitative analysis of the average detachment force between cells and gelatin functionalized colloidal tips using force spectrum method revealed the differences in cell adhesion characteristics, which cannot be achieved by traditional batch techniques. In the latter method, the experiment produced semi quantitative and average adhesion characteristics of a large number of cells. They are still limited by strict conditions and cannot highlight differences in adhesion within subpopulations of adherent cells. On the contrary, this AFM based method can perform reproducible quantitative studies on the adhesion characteristics of individual cells under common cell culture conditions, and can detect cell adhesion subgroups. These features meet key requirements in many fields, such as studying the migration ability of cancer cells.

#### 3.2.2. Wet State Peeling Test Fixture

At the engineering application level, the macroscopic pull-out method is one of the most commonly used techniques for evaluating the wet adhesion strength of viscoelastic materials. The testing device diagram is shown in Figure 3b. The general process of this method is to immerse the adhesive sample in a water tank and fully immerse it, and then press the adhesive body against the substrate surface with a set pretension force through the vertical movement of an automated force measuring platform. After the interface is fully in contact and subjected to constant temperature and time treatment, the adhesive body is pulled up from the substrate at the set pulling speed. At the same time, record the maximum peak force during the pulling process and standardize the peak force using the contact area to obtain the adhesion strength. For viscoelastic materials, the test results of the pull-out method strongly depend on the testing rate, which also reflects the rate dependence of the aforementioned viscoelastic dissipation mechanism. In recent years, Kim et al. [137] have further improved the automation and repeatability of wet state pull-out testing by utilizing commercial biomaterial mechanics testing platforms. With the help of three-dimensional micro nano pressure heads and the precision of cyclic control, previous studies have used these devices to measure the underwater switchable adhesion behavior of shape memory polymer micro column arrays. After microstructure assisted drainage of the water film, the polymer undergoes a complete cycle of heating softening, roughness adaptation, cooling shaping, and reheating recovery. This experiment achieved over 500 underwater pull-out tests and obtained data with good reproducibility. This series of improvements has elevated the underwater pull-out test from being only used for qualitative tape adhesion demonstrations in the past to the level of fracture mechanics research that can quantitatively track interface contact states.

Peel testing is a standard method for evaluating the adhesion performance of adhesives under actual stress modes, while the introduction of wet or underwater environments imposes special requirements on fixture design and testing specifications. For hydrogel soft adhesives, 90° peel test is a commonly used method in underwater adhesion research: after underwater bonding, the adhesive and the substrate peel off from the substrate at a constant angle and rate, and calculate the peel energy per unit width by recording the peel force. Yoon et al. [138] conducted a systematic evaluation of the effects of preload distance and stretching speed on the adhesion force of magnetic biomimetic suction cups. They found that the adhesion force tends to saturate with increasing preload, approximately 215 mN. Under fixed preload conditions, higher stretching speed can enhance the adhesion strength, which holds true for both dry and wet surfaces. Specifically, when the preload distance exceeds 0.2 mm, the adhesion force saturates at approximately 215 mN; under a fixed preload (0.4 mm), higher stretching speeds can enhance the measured adhesion force. Further research evaluated the adhesion performance on various substrates such as SiO_2_, as well as in dry, water/oil wet, and underwater environments. It was found that glass and Si adhered stronger under dry conditions, while PDMS and FEP exhibited higher adhesion in wet environments. This is consistent with the mechanism of weaker dry interface interactions and enhanced capillary action in wet conditions on low surface energy substrates. In terms of testing standardization, Chinese national standards have developed testing specifications for 90° and 180° peel strength of biomimetic adhesive materials, providing a methodological basis for comparable evaluation of underwater adhesion. Researchers at Lanzhou Institute of Chemical Physics have advanced the ability to regulate wet delamination at the device level. They integrated photo thermal responsive nanoparticles into biomimetic adhesive devices and found that nanoparticles can achieve reversible regulation of adhesion force through the delamination mechanism induced by mechanical deformation of the host material in both dry and wet environments. They have successfully been used for grasping and transferring underwater objects. This work demonstrates that delamination behavior is not only a characterization parameter, but can also serve as an engineering tool for actively regulating adhesion states.

#### 3.2.3. In Situ Observation of Optical Interference Total Reflection Imaging

Optical interference and suppressed total internal reflection techniques provide a non-invasive in situ characterization method for wet adhesion. The testing device diagram is shown in Figure 3c. This technology presses a glass lens with matched refractive index towards a transparent total reflection prism under controlled contact speed and load. By incident light at a specific angle on the back side of the prism, real-time capture of the contact radius and the discharge of water film at the interface can be achieved. When two surfaces are in complete contact, the attenuation of reflected light intensity indicates that the water layer is completely squeezed out. On the contrary, the light intensity in the non-contact area remains high, and this technique is suitable for studying the dynamic wetting dynamics of viscoelastic materials in water. For example, the contact expansion and drainage rate of polysiloxane hemispheres with different stiffness on hydrophobic and hydrophilic substrates. Research has found that when two hydrophobic surfaces approach underwater, although the theoretical adhesion is ultimately stronger, the actual firm contact must take longer to achieve due to the significant delay in water discharge. Compared to simply inferring the interface bonding state from the force displacement curve, this imaging technique directly reveals the thickness distribution of the water film and the geometric structure of residual water pockets, providing intuitive experimental evidence for explaining how the water retention effect affects the adhesion dynamics of viscoelastic materials.

The core challenge of wet adhesion lies in the discharge of interface water and the formation of contact, which occurs in thin liquid layers ranging from nanometer to micrometer scales, and traditional optical methods are difficult to distinguish in real time. The suppressed total reflection imaging provides a breakthrough in situ observation capability for this. Sun et al. [139] used high-resolution in situ infrared spectroscopy imaging to track the contact evolution process of soft elastic hemispheres with different stiffness and hard surfaces with different wettability underwater. They found that the water discharge rate in hydrophobic–hydrophobic contact was three orders of magnitude lower than that in hydrophobic–hydrophilic contact, revealing the deceleration effect of long-range hydrophobic attraction on contact formation. In terms of quantitative characterization, the same research group also developed an imaging technique based on FTIR spectroscopy, which can spatially resolve and quantify the thickness of nanoscale water puddles in underwater contact interfaces. This method was validated by comparison with Hertz contact theory and successfully used to characterize the drainage and plaque formation process of soft PDMS lenses approaching functionalized and original glass surfaces at different speeds. This technological route provides direct experimental evidence for understanding the dynamic bottleneck of interface water discharge in underwater adhesion, and also establishes a complete observation dimension from “force displacement curve” to “interface morphology evolution” for the design and evaluation of biomimetic adhesion surfaces.

#### 3.2.4. Summary

Overall, the micro nano scale testing of biomimetic wet adhesives has formed a multi-level method system from quantitative interfacial forces, macroscopic peeling evaluation to in situ interface imaging. Colloidal probe force spectroscopy achieved single-cell and even single-molecule level adhesion measurement through microsphere functionalization and dual scale testing, and revealed the enhancement mechanism of wet adhesion energy by hydrogen bond network disruption, solid–liquid phase transition, and interfacial fluidity [140,141,142,143]. Based on standard 90°/180° testing, the peeling and wet fixture technology combines preloading, tensile rate, and photothermal response to achieve reliable evaluation and reversible regulation of underwater adhesion strength; Optical interference and suppressed total reflection imaging were used to track the formation of underwater contact and the discharge of nanoscale water layers in situ. It was found that the drainage of hydrophobic interfaces was significantly slowed down, and the evolution of contact was controlled by wettability and material stiffness. The three complement each other, linking the force displacement curve, macroscopic peeling behavior, and interface water/contact morphology evolution, providing a multi-scale experimental basis for understanding the wet adhesion mechanism and guiding the design of underwater adhesives, biomimetic suction cups, and soft adhesion devices [144,145,146].

### 3.3. Summary

At present, the characterization of interface adhesion has evolved from a single macroscopic strength test to a multi-scale method system that complements macroscopic mechanics, micro nano force spectra, and in situ interface imaging. At the macro level, lap shear and tensile tests simulate actual construction through underwater gluing, pressing, and curing to evaluate the bearing capacity parallel and perpendicular to the bonding interface, and verify durability in combination with long-term immersion, hydrostatic pressure, and other environmental factors. The tape press back test quantifies the wet interface adhesion force, debonding work, and reversible recovery through the force displacement curve during the contact press separation process [147,148,149,150,151]. Peel testing evaluates the anti-peel performance of flexible tapes or adhesives on wet and rough surfaces through 90° and 180° configurations, and gradually moves towards standardization, multi environmental evaluation, and active regulation. At the micro nano level, atomic force microscopy colloidal probe force spectroscopy combines microsphere probes with force spectroscopy technology, utilizing functionalized probes, viscoelastic contact models, and dual scale measurements to quantify adhesion force, adhesion work, and relaxation behavior at the single-cell, single bacterial, and even single-molecule levels. Ultimately, the regulation of wet adhesion by interfacial water discharge, hydrogen bond network disruption, solid–liquid phase transition, and interfacial fluidity was revealed [152]. At the same time, wet peeling fixtures and automated pull-out platforms have improved the repeatability and cycling reliability of underwater testing. Optical interference and suppressed total internal reflection imaging are used to track in situ contact formation, water film drainage, and nanoscale water depression thickness. It was found that wettability and material stiffness significantly affect drainage and contact evolution. Overall, these methods are moving from a single strength index to multi index, multi-mode, multi environment, and in situ real-time evaluation, and are tending to establish underwater adhesion testing standards, develop visual testing platforms, and unify shear, tensile, adhesion, and peel data into interface constitutive models to promote practical applications [153].

## 4. Biomimetic Wet Adhesive

### 4.1. Synthesis Pathway of Wet Adhesive Inspired by Mussels

Inspired by this molecular strategy, researchers have constructed a series of functional materials. Among them, the clever unity of “wet adhesion” and “self-healing” performance is currently a research hotspot. Researchers have designed a network with dynamic covalent bonds or supramolecular interactions to mimic the dynamic properties of mussels in regulating the coordination between Fe^3+^ and catechol. When damaged, these dynamic keys achieve self-healing through rapid reassembly. Meanwhile, the high-density display of catechol groups not only releases water molecules, but also invades the adhesive interface, achieving high-strength interfacial bonding [154]. In order to overcome the limitation of relying solely on catechol to be easily oxidized, the scientific community further referred to the molecular strategy of cation–π interaction and hydrophobic microzone protection, and developed an environmentally tolerant adhesive based on functional polyurethane, hydrogel and other systems [155]. This type of new material, which combines wet adhesion and self-healing functions, has shown disruptive application prospects in fields such as biomedical engineering, flexible electronic sensing, and marine repair materials. This chapter will start with the physical and chemical molecular mechanisms of mussels, systematically explain the design principles of supramolecular dynamic networks, and explore how to achieve a dynamic balance between efficient wet adhesion and efficient self-healing through molecular strategy coordination.

#### 4.1.1. Synthesis of Peptides and Proteins

**Figure 4:**
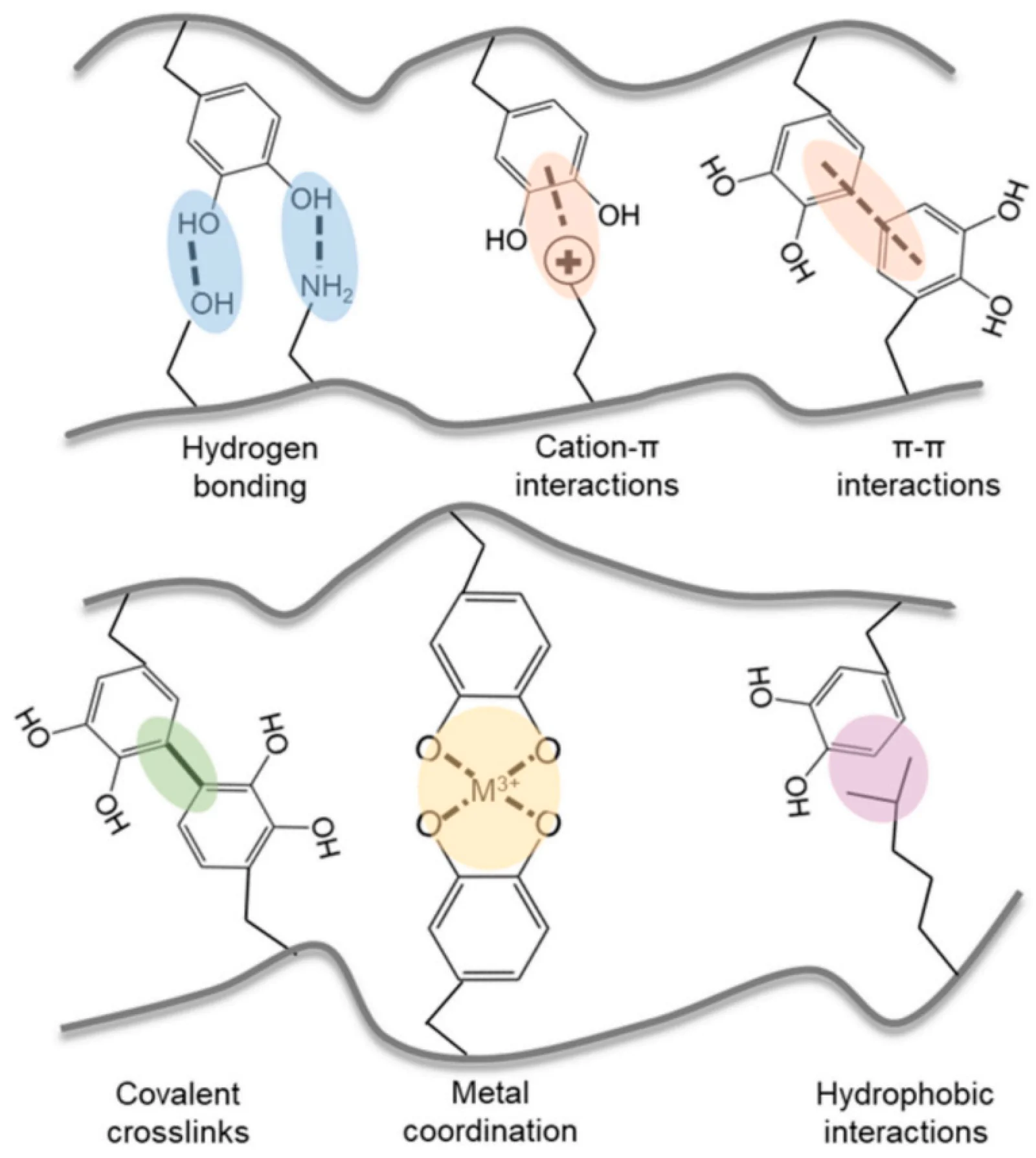
Schematic diagram of DOPA-mediated underwater adhesion [156]. The figure is not split. The scheme shows six key interactions: hydrogen bonding, cation–π interactions, π–π interactions, covalent crosslinks, metal coordination, and hydrophobic interactions. The catechol ring of DOPA provides hydrogen-bond donor/acceptor sites, a hydrophobic aromatic plane, and metal-coordination sites. Oxidation of DOPA to quinone enables covalent crosslinking. These interactions act synergistically to displace interfacial water, promote surface contact, and strengthen both interfacial adhesion and cohesive network formation.

Marine mussels achieve firm adhesion to various surfaces in wet conditions through their footplate, and the molecular basis of this ability lies in the secretion of various byssus proteins, especially Mfp-3 and Mfp-5 located at the adhesion interface. The prominent feature of these two proteins is the high proportion of DOPA. The catechol side chains of DOPA can form reversible bonds with the substrate through hydrogen bonding, metal coordination, and other means (Figure 4), and can also participate in covalent crosslinking after oxidation to enhance the strength of the substrate. This cleverly unifies the two functions of interface adhesion and cohesive solidification [156]. It is precisely this precise molecular design that has given birth to the biomimetic strategy in mussel inspired wet adhesives that is closest to natural logic: the synthetic peptide pathway. The core idea of this strategy is to directly replicate the sequence and structure of natural adhesive proteins, and to precisely construct peptide molecules containing DOPA and its synergistic residues (such as lysine and arginine) through chemistry or biosynthesis. The simplified short peptide model constructed using solid-phase peptide synthesis technology in the early days not only confirmed the adhesive contribution of individual DOPA residues at the nanoscale, but also revealed the synergistic mechanism of cationic residues’ release of catechol through charge repulsion or competitive coordination, thereby promoting intrinsic crosslinking. On this basis, researchers further synthesized block peptides that mimic the modular features of natural proteins [157]. Among them, DOPA is densely distributed on one end to be responsible for interface adhesion, and the other end is rich in hydrophobic or cationic motifs to induce liquid–liquid phase separation, successfully constructing a condensed adhesive that can spontaneously aggregate and rapidly cure in wet state. With the integration of synthetic biology technology, recombinant protein expression systems can convert tyrosine in situ into DOPA, directly producing full-length adhesive proteins and even block hybrid peptides. This not only increases sequence complexity, but also endows materials with intelligent properties such as self-assembly and stimulus response [158].

The synthetic peptide pathway provides an ideal platform for analyzing the structure–activity relationship of wet adhesion at the molecular level, and with its inherent biodegradability and cell compatibility, it has shown promising application prospects in the biomedical field. Currently, the challenges faced in this field mainly focus on the high cost of synthesizing and purifying long-chain peptides, as well as the limited efficiency of unnatural amino acid incorporation in recombinant expression. Inspired by mussel foot protein and chitosan based adhesives, Lu et al. [159] used partially NH_2_—protected chitosan as an initiator to synthesize a series of chitosan grafted peptide copolymers through ring opening polymerization of peptide compounds, which have both good biodegradability and low cytotoxicity; the lap shear test showed that its maximum strength against pig skin and aluminum sheet reached 195.97 kPa and 3080 kPa, respectively, and the tensile bonding strength of the bone was 642.70 kPa. In vivo experiments in rats confirmed its excellent hemostatic effect and can promote the healing of skin wounds and fractures. In addition, Jing et al. [160] prepared non-swelling protein adhesives by electrostatic complexation of super charged peptides with surfactants containing DOPA (or azobenzene). This adhesive has better environmental adhesion ability than commercial cyanoacrylate, and its underwater adhesion is also better than other reported protein based adhesives. In vitro and in vivo experiments have verified its wound sealing and healing performance. Although the synthetic peptide pathway provides an ideal platform for analyzing the wet adhesion structure–activity relationship, the high cost of long-chain synthesis and purification, as well as limited efficiency in incorporating unnatural amino acid, remain current challenges.

In response to the strong adhesion under harsh conditions, Wang et al. [161] developed an in vitro mussel inspired adhesion system, which prepared adhesive peptides rich in DOPA and lysine through two-step enzymatic catalysis of catalytic polymerization and tyrosinase mediated hydroxylation. In addition, by utilizing click chemistry to covalently immobilize enzymes onto functionalized diatom cones, high tyrosine conversion (86.7%) and six cycle activity maintenance of approximately 80% were achieved. The peptide was grafted onto a polyglutamic acid backbone and subjected to DOPA induced phase separation. The resulting condensed acid salt had a shear strength of up to 31.8 MPa and exhibited stable adhesion in air, water, oil, organic solvents, and within the temperature range of −196 °C to 150 °C. In another strategy, Berger et al. [162] prepared brush-like protein mimetics by ring opening metathesis polymerization of norbornene based peptide monomers, which have better solid surface adhesion than natural proteins and can be used for live cell fixation. Tobias et al. [163] revealed that during the formation of blue mussel feet, iron and vanadium ions are stored in intracellular metal storage particles containing catechol biomolecules, and mix with secretory vesicles in microfluidic-like channels to form protein metal bonds, providing new insights for the design of metal polymers and adhesives. These studies have jointly promoted the development of high-performance mussel inspired adhesives and demonstrated broad prospects in hemostasis, soft tissue repair, and extreme environmental adhesion. Solid-phase peptide synthesis (SPPS) has irreplaceable advantages in sequence and side chain control for precise simulation of short sequences.

Yang et al. [164] synthesized two types of mussel mimetic peptides using Fmoc DOPA (Acetylide)—OH protection strategy, and the resulting products have completely natural peptide structures that can flexibly control the amount and sequence arrangement of catechol. Tang et al. [165] achieved adjustable grafting of catechol groups by coupling lysine enriched peptides with acetone protected dihydroxy hydrogenated cinnamic acid. SPPS is limited by peptide chain length and cost, making it more suitable for short sequence active domains and mechanism validation, while full-length protein and large-scale preparation still rely on recombinant expression and enzyme catalysis. The current research trend is shifting from single pathway optimization to multi pathway integration: recombinant expression provides a soluble protein backbone, enzyme catalysis completes DOPA hydroxylation, SPPS constructs short peptide probes or functional fragments, forming a complete chain from molecular design to large-scale preparation. In the future, it will still be necessary to address the quantitative structure–activity relationship between DOPA oxidation control, DOPA content and adhesion strength, as well as customized production issues for different scenarios such as underwater bonding and tissue sealing.

The synthetic peptide pathway is the most natural biomimetic strategy inspired by mussels in wet adhesives. Its core lies in accurately reproducing functional sequences containing DOPA and synergistic residues through chemistry or biosynthesis. The early solid-phase synthesis of short peptide models not only quantified the nano adhesion contribution of individual DOPA residues, but also revealed the synergistic mechanism of cationic residues promoting host crosslinking through charge repulsion. On this basis, the design of block peptides introduces modular features, with DOPA densely distributed at one end responsible for interface binding, and hydrophobic or cationic motifs enriched at the other end to induce liquid–liquid phase separation, thereby obtaining a condensed adhesive that can spontaneously aggregate and quickly solidify in a wet state. In response to the challenges of high cost of long-chain synthesis and purification, as well as low efficiency of unnatural amino acid incorporation, a multi-step enzyme catalytic strategy has been developed recently. Through tyrosinase mediated hydroxylation, adhesive peptides rich in DOPA and lysine are efficiently prepared, and click chemistry immobilized enzymes are used to achieve high conversion rates and excellent cycling stability. After grafting onto the polyglutamic acid backbone, the obtained peptide undergoes DOPA induced phase separation to form a condensed acid salt. Its shear strength reaches several tens of megapascals and it can maintain stable adhesion in air, water, oil, organic solvents, and a wide temperature range (−196 °C to 150 °C). These improvement strategies around the synthesis of peptides and their derivatives have jointly promoted the development of high-performance wet adhesives, showing promising application prospects in hemostatic sealing, soft tissue repair, and extreme environmental bonding.

A closer look at the peptide route, however, reveals an unresolved structure–performance tension that is rarely discussed. The DOPA content within a peptide sequence does not scale linearly with adhesion: below a threshold density, catechol residues cannot displace the hydration layer efficiently; above it, spontaneous oxidation consumes the interfacial catechol before contact is established, and the oxidized quinone preferentially forms intra-chain cross-links that embrittle the plaque rather than bond the substrate. The spatial arrangement of DOPA relative to cationic residues matters as much as their absolute count. Block-segregated designs, in which lysine and DOPA occupy distinct domains, outperform statistically randomized sequences by a factor of two to three, yet this design rule is still derived from a handful of model peptides rather than a systematic sequence library. It is also worth emphasizing that the headline 31.8 MPa obtained by enzyme-catalyzed DOPA-rich peptides was measured on rigid, clean substrates over an extreme temperature window, whereas on hydrated soft tissue the same peptide chemistry drops to the 10 to 100 kPa range. Comparing these numbers as if they described the same material obscures the substrate-dependent failure mode. The peptide route therefore remains unmatched as a mechanistic discovery platform, but its economic and translational ceiling, including solid-phase synthesis cost, low-yield tyrosinase hydroxylation, and batch-to-batch variability inherent in recombinant expression, means it is unlikely to become the primary manufacturing route for structural adhesives; its realistic near-term role is in high-value biomedical and surface-coating niches.

#### 4.1.2. Modified Polymer

By introducing catechol groups into the polymer skeleton to simulate mussel adhesion proteins, the intrinsic properties of the polymer can be fully utilized and the distribution of functional groups can be regulated to achieve synergistic adhesion and processing performance. However, in practical applications, there are bottlenecks such as solidification control, interface drainage, and pH adaptability. Single catechol are difficult to eliminate interstitial water, and most systems are only efficient under alkaline conditions, limiting biomedical applications. Therefore, current synthesis strategies tend towards multifunctionality, such as introducing dynamic crosslinking to regulate solidification, designing amphiphilic chain segments to enhance drainage, developing pH independent linking chemistry, and exploring biomass based catechol monomer preparation to enhance sustainability. The modified polymer pathway is breaking through the wet adhesion problem through molecular fine design and expanding to fields such as coatings and energy devices. Sukhmanjot et al. [166] designed catechol containing elastomers through coumarin ([2+2]) ring crosslinking. Combined with spectroscopy, it was confirmed that catechol relies on multiple hydrogen bonds to contribute to dry/wet adhesion, but cannot exclude interstitial water on its own. Hydrophobic groups need to cooperate with dehydration to provide a basis for the wet adhesion mechanism. Du et al. [167] efficiently prepared catechol copolymers from C-lignin through depolymerization, derivatization, and copolymerization, achieving ultra-high adhesion of 2.0 MPa and 8.1 MPa on hydrophobic PTFE and steel in seawater, respectively, achieving atomic economy conversion. Soohwan et al. [168], aiming at the limitation that oxidized catechol is only suitable for alkaline conditions, used aldehyde modified hyaluronic acid to realize catechol dual-mode (amide secondary amine) connection, rapidly gel under acid/neutral pH and maintain strong adhesion and biocompatibility, expanding the synthesis strategy of pH universal catecholamine chemistry. Asila et al. [169] prepared viscous hydrogel by covalent combination of catechol and levorotatory. The adhesive strength to hydrated pigskin reached 42.17 kPa (three times of super fibrin glue). At the same time, it promoted blood coagulation and accelerated skin healing in rats, and the immune response was low, which was suitable for hemostasis and repair. Yu et al. [170] first used catechol modified ionomers for anion exchange membrane fuel cells. After 2500 h in 2 M NaOH at 80 °C, the conductivity of the modified polymer only slightly decreased, and the single cell performance improved by 34%. It operated stably for over 300 h at 0.4 A/cm^2^. The combination of theory and experiment revealed the molecular adhesion mechanism between catechol and Pt catalyst and its regulation of the microstructure of the catalyst layer, providing a new path for the molecular design of membrane electrode ionomers.

Within the modified-polymer family, the structure–performance relationship is governed by three coupled levers that are still poorly decoupled in practice: catechol grafting density, backbone flexibility, and cross-link density. Increasing catechol loading raises interfacial adhesion but simultaneously accelerates oxidation-induced cohesion loss and swelling-driven plasticization in wet service; stiffening the backbone to resist swelling reduces chain mobility and therefore impairs the wet-out and drainage step that catechol chemistry depends on [171]. The result is that systems reporting MPa-scale adhesion on steel typically rely on rigid thermoset matrices in which catechol is a minority side group, whereas soft hydrogels carrying a high catechol fraction rarely exceed 50 to 200 kPa on tissue. This distinction is often blurred in narrative reviews: catechol-modified polymer spans materials as structurally different as an epoxy thermoset and a polyurethane hydrogel, and their adhesion cannot be meaningfully ranked by a single number [121]. A further limitation is that most catechol grafting is performed by random copolymerization, so the spatial distribution of interfacial groups cannot be controlled at the nanoscale the way natural Mfp-3/5 concentrates DOPA precisely at the plaque–substrate interface. Directed assembly remains underexplored relative to its mechanistic promise.

### 4.2. Synthesis Pathway of Wet Adhesive Inspired by Barnacles

The current mainstream synthesis strategy is to mimic the liquid–liquid phase separation characteristics of barnacle cement protein when it encounters seawater. Specifically, resistant to dilution “coagulation layer” adhesives are prepared through molecular design. A typical representative is the team led by Yi et al. [19] from South China University of Technology, which modified the hydrophobic end groups of polypropylene glycol (PPG) by grafting butyl groups, resulting in spontaneous phase separation upon contact with water. This type of cohesive layer combines super spreading and strong resistant to dilution properties. After UV curing, the adhesion strength reaches 479 kPa and can withstand a burst pressure of 436 kPa. It has been successfully used in animal experiments for sealing gastric perforation and intestinal wounds.

Another idea is to directly imitate the amino acid composition of barnacle protein, copolymerize cationic monomer (such as ATAC) and aromatic monomer (such as PEA), and build a hydrogel network rich in π–π stacking and cation–π interaction, so as to enhance cohesion and interfacial adhesion. In addition, the researchers also integrated the bionic principle with other technologies, such as the preparation of chitosan based hydrogel by combining solvent exchange technology to achieve on-demand debonding and controllable adhesion. Furthermore, the design of amphiphilic polyurethane adhesives utilizes the crystallinity of hydrophilic/hydrophobic segments to drive molecular migration, resulting in enhanced adhesion performance in both underwater and oil environments.

The barnacle-inspired route offers a conceptual advantage that the DOPA route does not: by sequestering adhesive chemistry inside a water-immiscible coacervate, it sidesteps the oxidation conflict entirely and tolerates seawater dilution without a pH trigger. Its weakness, however, is that the synthetic versions reported to date reproduce only one level of the natural design. Natural barnacle cement relies on hierarchical assembly: interface proteins (cp19k/cp20k/cp43k) anchoring to the substrate, bulk proteins (cp52k/cp100k) assembling into amyloid β-sheets, and enzymatic cross-linking catalyzed by lysyl oxidase and transglutaminase; synthetic mimics, by contrast, typically reproduce only the hydrophobic end-group modification or a cationic/aromatic copolymer network [172]. The consequence is that reported lap-shear strengths cluster at 0.2–0.5 MPa, roughly an order of magnitude below mussel-inspired epoxy systems, and the curing rate is often slow because the amyloid-like self-assembly step cannot be accelerated without also triggering premature gelation. Whether this gap is intrinsic to the phase-separation strategy or merely reflects the immaturity of synthetic protein-mimetic design remains an open question. Critically, the barnacle route’s strongest selling point, reversible debonding and repositionability, is also its Achilles’ heel for permanent bonding: the same hydrophobic microphase separation that resists dilution also limits the ultimate cross-link density, so systems engineered for on-demand release tend to sacrifice long-term creep resistance [173].

### 4.3. Synthesis Pathway of Wet Adhesives Inspired by Sandcastle Worms

Inspired by the protein glue secreted by sandcastle worms that can firmly bond sand particles underwater, the synthesis of biomimetic wet adhesives focuses on replicating the key chemical and structural features of their natural glue. Research has shown that the core component of sandcastle worm glue is a polyelectrolyte protein with opposite charges, which forms a liquid precursor called a “composite condensate” through electrostatic interactions. This process is crucial for the stable transportation and solidification of glue underwater. Based on this principle, researchers have designed anionic and cationic peptides that simulate the amino acid composition of natural adhesive proteins. These peptide chains introduce key functional groups, such as phosphate groups from phosphoserine (PSER) and catechol groups from DOPA. The former provides negative charge and adhesion function, while the latter endows excellent interfacial adhesion with its strong metal coordination and hydrogen bonding ability.

In terms of specific synthetic pathways, the current mainstream methods can be divided into two categories: one is chemical synthesis based on amino acid monomers, and the other is grafting adhesive functional groups onto the polymer backbone. The most commonly used method for peptide biomimetic adhesives is peptide solid-phase synthesis, which can efficiently and accurately synthesize specific sequences of anionic and cationic peptides with a purity of over 95%. In order to obtain high molecular weight copolymer peptides that are closer to natural proteins, Li et al. [174] synthesized copolymer peptides by N-carboxycyclic anhydride (NCA) ring opening polymerization (ROP). This method can flexibly synthesize copolymer peptides with adjustable phosphorylation degree (negativity) and DOPA content (adhesiveness). Another approach is not limited to peptides, but involves grafting functional molecules such as catechol and phosphate groups onto synthetic polymer frameworks such as epoxybutane to obtain water-soluble wet adhesives. In addition, Dong et al. [175] also developed a simpler strategy to realize the functionalization of wet hydrogels in one step by synthesizing dibutylamine DOPA lysine DOPA (DbaYKY) tripeptide and connecting it to various polymer chains as a terminal group.

The final key step of synthesis is to simulate the composite coagulation process of the sandcastle worm secreting glue. By mixing synthesized anionic and cationic peptides or polyelectrolytes in a certain proportion, they will spontaneously separate through electrostatic interactions, forming a thick, water-insoluble, low surface tension, and shear thinning complex. This liquid aggregate is easy to apply accurately underwater and can then be cured through enzymatic crosslinking or pH/ionic strength changes to form a strong bond. Research has shown that by adjusting parameters such as the molar ratio, pH value, and ionic strength of peptides, the coagulation and adhesion properties can be finely tuned. For example, a mixture of anionic and cationic peptides in equal proportions can increase the adhesion force by nearly twice that of a single component, reaching 347.81 kPa [176]. This biomimetic synthesis pathway provides a new reference for the development of next-generation wet adhesives for fields such as tissue engineering.

Polyelectrolyte complex coacervation is often described as rapid-curing, but its structure–performance window is narrower than this label suggests. Equimolar mixing of oppositely charged peptides approximately doubles the adhesion relative to either component alone. However, even a slight deviation from stoichiometry precipitates either a soluble excess polyelectrolyte or a highly aggregated, non-spreadable complex. Its processing latitude is therefore far tighter than that of step-growth epoxy curing [177]. The phosphorylated-serine motif is often touted as a DOPA-independent alternative, but it has its own limitations: phosphate-metal coordination is strong on calcium-rich mineral surfaces but comparatively weak on oxides such as glass or titanium, and phosphate esters are hydrolytically less stable than catechol-quinone adducts over multi-year immersion. In terms of performance, the lap-shear strengths of coacervate adhesives reported to date cluster in the 50–500 kPa range, comfortably above soft-tissue hydrogels but well below rigid thermosets; their genuine advantages lie in injectability, microscale phase-separated morphology, and compatibility with 3D printing. Therefore, positioning the sandcastle-worm route as a direct competitor to mussel-inspired epoxy systems for structural bonding is misleading; its defensible niche is minimally invasive, in situ forming, or patterned adhesive applications, where a pre-cured rigid joint cannot be delivered.

### 4.4. Summary

The comparison of synthetic pathways for biomimetic wet adhesives is shown in Table 3. Biomimetic wet adhesives inspired by mussels, barnacles, and sandcastle worms have their own unique molecular strategies, synthesis pathways, and performance orientations. The biomimicry of mussels relies on the catechol chemistry of DOPA and follows the temporal adhesion logic of “reversible recognition and irreversible locking”. It constructs multifunctional materials through solid-phase peptide synthesis, enzyme catalyzed modification, or polymer grafting. Although it has excellent adhesion strength and both self-healing and biocompatibility, it is limited by the inherent conflict between catechol oxidation and interface adhesion, as well as pH sensitivity. The biomimetic design of barnacles focuses on the condensed layer formed by liquid–liquid phase separation, and utilizes hydrophobic modification or amphiphilic copolymerization design to endow the adhesive with resistant to dilution, resistant to erosion, and reversible debonding abilities, making it suitable for complex dynamic environments. However, the curing rate and large-scale preparation are still bottlenecks. The biomimetic Sandcastle worm utilizes the principle of polyelectrolyte composite coagulation, which drives the assembly of proteins or peptides with opposite charges through electrostatic driving. Through metal ion coordination and enzymatic cross-linking, it achieves rapid underwater solidification. Its adhesion performance can be flexibly adjusted by ion environment and is suitable for additive manufacturing. However, the precise control of the coagulation process and long-term service stability still need to be deepened [178]. All three types of strategies have made significant progress in molecular mechanism analysis and functional integration. However, they still face common challenges in terms of persistence in dynamic wet environments, quantitative balance of multifunctional collaboration, and engineering transformation. It is urgent to achieve new breakthroughs through intelligent response design and data-driven optimization.

## 5. Application of Biomimetic Adhesives

Biomimetic wet adhesives inspired by marine organisms exhibit unique interfacial adhesion properties in humid and even completely submerged environments. Currently, the application research of this type of material is advancing in depth along three main directions: wearable electronic devices, industrial facilities and marine engineering restoration, and biomedical research. In the field of wearable technology, researchers have utilized the adhesion strategies of mussels, barnacles, and sandcastle worms to design a sensing interface that combines high stretchability, conductivity, freeze resistance, and self-healing capabilities, solving the core bottleneck of signal acquisition instability in flexible electronics under dynamic wet physiological environments. In marine and industrial scenarios, biomimetic adhesives are endowed with comprehensive properties such as rapid underwater curing, high bonding strength, long-term hydrolysis resistance, and anti-fouling and fire resistance to meet the needs of structural bonding and sealing repair in complex water environments. In the biomedical field, this type of material exhibits significant advantages over traditional sutures and commercial products in wound closure, infected wound healing, tissue regeneration, and implantable bioelectronic interfaces due to its excellent wet tissue adhesion, hemostatic efficiency, antibacterial ability, and biocompatibility.

### 5.1. Wearable Devices

**Figure 5:**
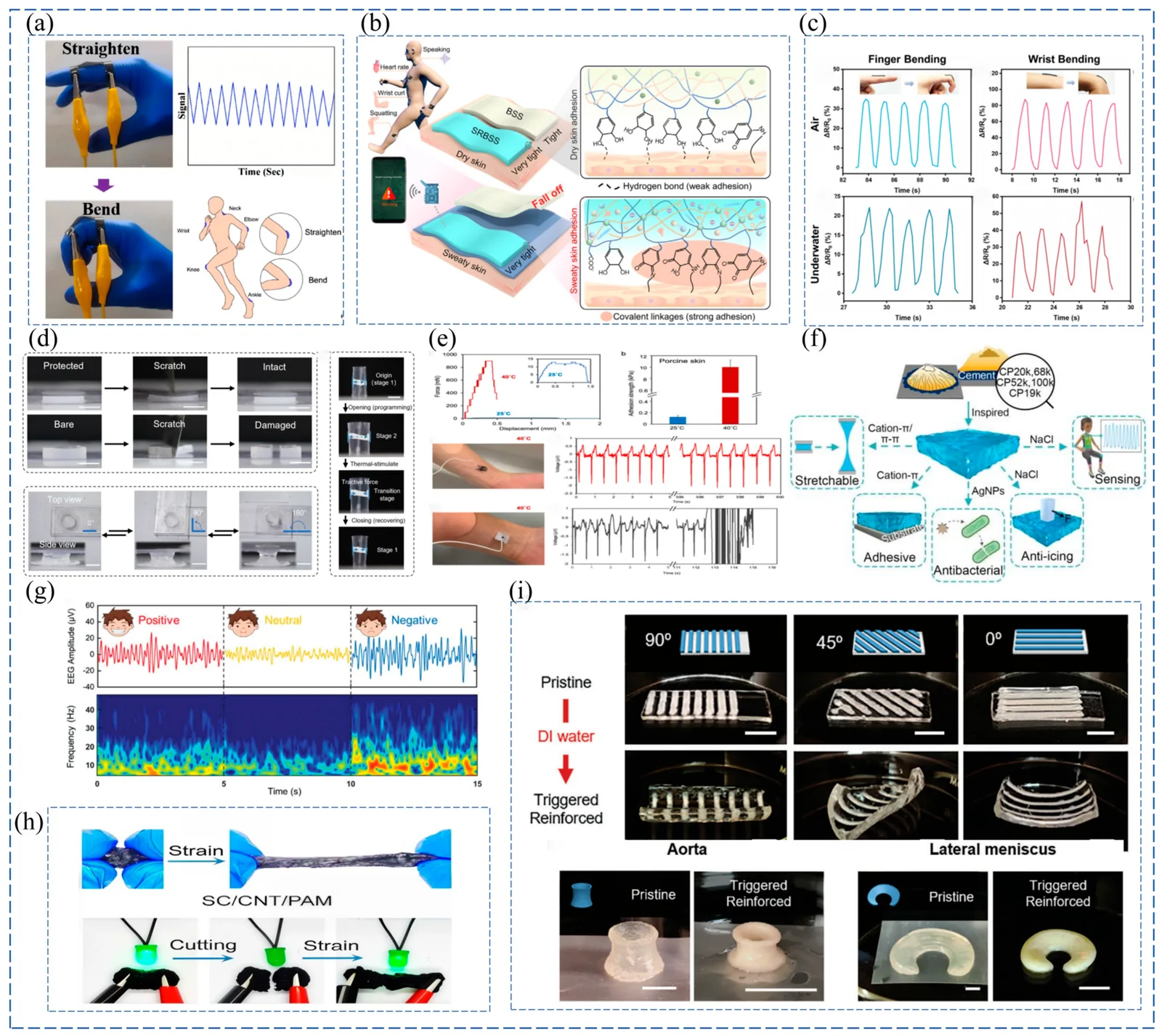
Applications of biomimetic adhesives in wearable devices. (**a**) Mussel-inspired dry-state polylactic acid/liquid metal (PLA-LM) 3D bead-like adhesive for wearable motion sensing [179]. (**b**) Mussel-inspired sweat-resistant bioelectronic skin sensor for remote disease early warning [180]. The sensor exhibits strong adhesion, 800% stretchability, instant self-healing in less than 1 s, and −40 °C freeze resistance. The panel shows the sensor design, operating principle, and structure. (**c**) Barnacle-inspired MXene/PHMP hydrogel sensor for air and underwater strain/pressure sensing [181]. Hydrophobic monomer design resists interfacial water invasion and provides repeatable wet adhesion on glass, PET, and pigskin. (**d**) Mussel-inspired hydrogel−plastic hybrid with robust bonding [182]. The system can collect electrical signals on human skin for more than 10 min and demonstrates scratch resistance, flexible connection, and temperature-controlled artificial shell closure. (**e**) Mussel-inspired thermoswitchable underwater adhesive (TSA hydrogel) based on lower critical solution temperature (LCST) behavior and mussel molecules [183]. Reversible adhesion on glass, titanium, aluminum, and Teflon is regulated by more than 1000 times, and gentle detachment occurs at low temperature to avoid tissue damage. The panel compares ECG monitoring with commercial hydrogel electrodes. (**f**) Barnacle-inspired hydrogel based on cation−π interaction, hydrophobic interaction, and ionic coordination [184]. It can attach directly to human skin to detect weak physiological signals. (**g**) Mussel/barnacle dual-inspired self−adhesive/hydrophobic bilayer hydrogel (AHBH) [185]. As an electrode/skin interface, it collects electrophysiological signals under vibration and sweating and is integrated into a portable headband for EEG−based emotion classification. (**h**) Sandcastle-worm-inspired xerogel [186]. It rapidly absorbs water and self-crosslinks into a hydrogel within 8 s. The material is recyclable and reconfigurable. The panel shows the hydrogel illuminating an LED in a closed circuit. (**i**) Sandcastle−worm−inspired printable tough adhesive (PTA) [187]. 3D printing and in situ mechanical enhancement enable patterned biological adhesion and 4D soft robot programming. The panel shows 3D CAD models, printed structures, and shape changes when immersed in deionized water.

In the emerging field of wearable electronics, stable interface adhesion between devices and engineering substrates is one of the core challenges. Traditional adhesives often fail quickly in humid, dynamic, or complex physiological environments, while organisms such as mussels, barnacles, and sandcastle worms in nature, with their unique interface chemistry and structural design, have achieved strong and reversible adhesion underwater, providing rich inspiration for the development of artificial adhesive materials. In recent years, researchers have combined biomimetic strategies with functional materials, resulting in a series of new systems that combine high adhesion strength, conductivity, self-healing, and environmental adaptability. Zhou et al. [179] combined mussel biomimicry with mechanical interlocking mechanism to prepare PLA-LM 3D bead structure adhesive, which achieved excellent properties of shear strength of 2.7625 MPa and peel strength of 290 kPa. The shear strength was further increased by 10.77% under solar irradiation using the EGaIn photothermal effect (Figure 5a). Its self-adhesive sensor demonstrates the strain electrical signal detection function, representing a new paradigm of dry adhesive that combines environmental responsiveness and adhesion enhancement. The anti-sweat bioelectronic skin sensor developed by Li et al. [180] exhibits strong adhesion, 800% stretchability, instant self-healing in less than 1 s, and −40 °C freeze resistance in complex physiological environments (Figure 5b). He et al. [181] MXene/PHMP hydrogel inspired by barnacle protein effectively resists the invasion of water molecules at the interface through hydrophobic monomer design, and exhibits repeatable wet adhesion on glass, PET, pigskin and other substrates (Figure 5c). The conductivity reaches 0.016 S/m, the strain coefficient is 7.17 (200% −500% strain), and the pressure sensitivity is 0.63 kPa^−1^ (0–70 kPa), which together demonstrate the applicability of wet/dry multi scenario.

In terms of reversible adhesion and underwater adaptability, Wen et al. [182], inspired by the mussel rigid soft synergistic structure, achieved a bonding strength of 1.52 MPa and an interface toughness of 1450 J·m^2^. Its temperature response switch ratio was more than 1000 times. It can collect electrical signals on human skin for more than 10 min, providing a new idea for intelligent robots and temperature control interfaces (Figure 5d). The thermally switchable underwater adhesive (TSA hydrogel) developed by Abe et al. [183] uses the LCST behavior of the thermal responsive polymer and mussel molecules to achieve the reversible regulation of the adhesive strength on glass, titanium, aluminum and Teflon substrates by more than 1000 times, and gently detaches at low temperatures to avoid tissue damage (Figure 5e). Fu et al. [184], inspired by the barnacle structure, realized 1430% extensibility, 22.8 kPa adhesion strength and 18.5 mS/cm conductivity through cation–π interaction, hydrophobic interaction and ionic bond coordination, which can directly attach to human skin to detect weak physiological signals (Figure 5f). Inspired by the adhesion mechanism of mussels and barnacles, Yang et al. [185] developed a self-adhesive/hydrophobic double-layer hydrogel (AHBH) with an adhesion force of 59.7 N/m and a hydrophobic layer with a water contact angle of 133.87 ° after 0.5 h treatment. As an electrode/skin interface, this material can reliably collect electrophysiological signals under vibration, sweating and other conditions, and is integrated into a portable headband for EEG based emotion classification (Figure 5g). These works significantly expand the spatiotemporal controllability and functional integration of biomimetic adhesive materials. Tang et al. [186], a miniature dry gel inspired by the sand castle worm, can quickly absorb water and self-crosslink to generate hydrogel in 8 s. The adhesion energy is higher than 350 J·m^−2^, the conductivity is higher than 2.5 S·m^−1^, and it is recyclable and reconfigurable (Figure 5h). Ma et al. [187] were inspired by the sandcastle worm to print tough adhesive (PTA), which achieved patterned biological adhesion and 4D soft robot programming in physiological environments through 3D printing and in situ mechanical enhancement (Figure 5i). These works significantly expanded the spatiotemporal controllability and functional integration of biomimetic adhesive materials.

The application comparison of biomimetic adhesives in the field of wearable devices is shown in Table 4. Although some systems mention anti-swelling or anti-perspiration design, the volume expansion and mechanical degradation of most hydrogels in long-term immersion or high humidity environment still far from meeting practical requirements. Although temperature responsive reversible adhesion provides separation convenience, its response rate, hysteresis effect and performance loss rate after multiple cycles have not been fully evaluated. More importantly, biosafety evaluations generally only focus on cytotoxicity or short-term skin contact, and there is a severe lack of in-depth research on degradation products, chronic inflammatory reactions, and long-term compatibility in vivo. Additionally, there is a lack of clear data on the recovery efficiency of interface resistance and mechanical strength after reattachment of reconfigurable systems.

In addition, most studies simply stack multiple functions such as conductivity, self-healing, and high stretchability, but rarely explore the inherent constraints between each performance. The differentiated requirements for performance priority in different scenarios in practical applications have not been addressed in material design. In the future, it is urgent to establish standardized testing protocols for dynamic physiological environments and promote a paradigm shift from “multifunctional integration” to “optimization of working condition adaptability” in order to accelerate the effective transformation of laboratory results into practical wearable electronic and biomedical devices.

### 5.2. Industrial Facilities and Marine Restoration

The rapid development of ocean engineering, ship equipment, and underwater infrastructure has put forward an urgent demand for adhesive materials that can quickly solidify, remain stable for a long time, and have high strength in seawater environments. Traditional adhesives often fail underwater due to interface water film isolation, curing obstruction, or poor corrosion resistance, while marine organisms can achieve strong adhesion in humid or even fully underwater environments, providing a natural template for the molecular design of new underwater adhesives. In recent years, researchers have developed a series of biomimetic epoxy resins, elastomers, and composite coatings by simulating the adhesive protein structure, functional group composition, and cross-linking mechanism of these organisms, achieving significant breakthroughs in adhesive strength, curing speed, environmental resistance, and large-scale preparation.

**Figure 6:**
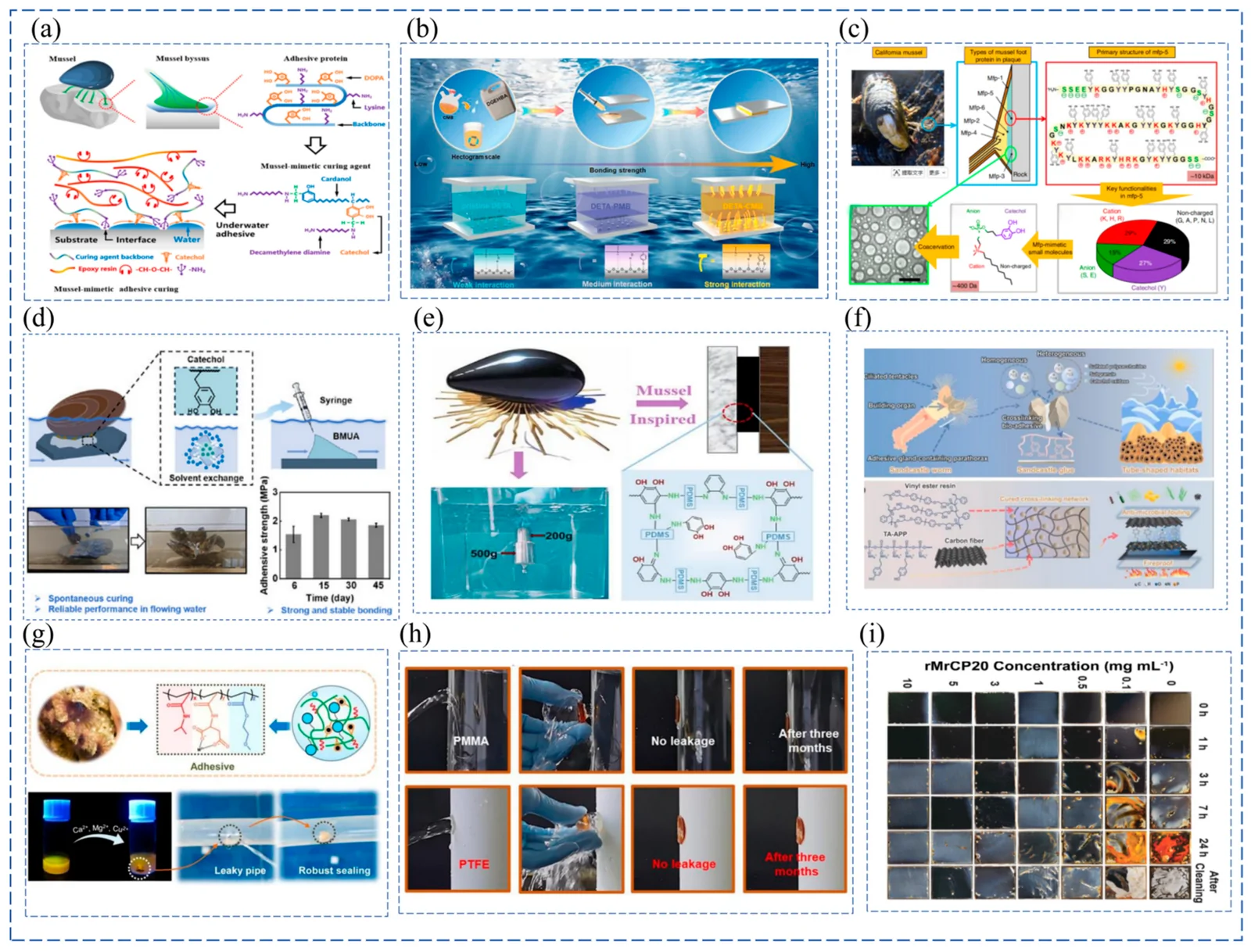
(**a**) Mussel−inspired fast−curing epoxy adhesive for offshore equipment and bridge repair [188]. Cashew phenol and catechol are incorporated into the main chain via the Mannich reaction, and decyldiamine is added to mimic protein amino acids. Ions in seawater participate in non−covalent bond formation. Arrows indicate the synthesis/curing/bonding process. (**b**) Mussel−inspired catechol−based Mannich base curing agent (CMB) prepared by a one-pot condensation reaction for reinforced metal bonding interfaces [189]. After bonding with hydrogenated bisphenol A epox. The catechol group shows good durability under hydrothermal conditions. (**c**) Low−molecular−weight zwitterionic surface-wetting adhesive inspired by mussel adhesive proteins [190]. The adhesive self-assembles into a strong adhesive layer with a thickness of less than 4 nm and an adhesion energy of approximately 50 mJ/m^−2^, making it suitable for nanofabrication and nanoprocessing. (**d**) Mussel−inspired underwater adhesive (BMUA) prepared by radical solution copolymerization of DOPA, methacrylic acid, N−isopropylacrylamide, and methyl methacrylate [191]. Solvent exchange triggers hydrogen bonding and hydrophobic crosslinking. It also shows good biocompatibility and hemostatic sealing performance. (**e**) Mussel−inspired, catalyst-free, environmentally friendly silicone adhesive used as an underwater sealant [192]. Extremely fast underwater curing occurs in the presence of hydrogen peroxide, effectively blocking leaks. (**f**) Biomimetic multifunctional carbon fiber composite material inspired by sandcastle worms, used for antibacterial, anti−fouling, and flame retardant applications [193]. The adhesive polymer interface contains casein and ammonium phosphate motifs. The phenolic hydroxyl group of casein enhances the chemical affinity between ammonium polyphosphate, vinyl ester resin, and carbon fiber, while providing broad−spectrum antibacterial and anti-fouling properties. Working together with ammonium phosphate, it achieves ultra−high fire safety and is suitable for surface protection of marine facilities. (**g**) A copolymer/Mn ^+^ biomimetic adhesive inspired by sandcastle worms based on temperature enhanced condensation [194]. The copolymer contains hydrophobic, bidentate anions, and thermosensitive segments. Ca^2+^/Mg^2+^/Cu^2+^ ion induced aggregation and temperature enhanced dehydration. This adhesive can bond glass, silicon, polytetrafluoroethylene (PTFE), and animal bones, and can seal leaking pipes or containers underwater. (**h**) An adhesive inspired by barnacles used for sealing barrel holes and waterproofing [196]. The photo shows that it has strong adhesion on PMMA and PTFE substrates. This board demonstrates the adhesive’s ability to bond multiple surfaces and prevent water leakage. (**i**) The corrosion resistance of low carbon steel under simulated high salt ocean conditions using recombinant barnacle gum protein (rMrcP20) [195]. The optical image shows the steel surface treated with different concentrations of rMrcP20 and soaked for different times. This protein forms a stable protective layer with the iron ions released from the steel substrate, thereby increasing the coating impedance and delaying corrosion.

Inspired by the dopamine structure and amino acid composition of mussel adhesion proteins, Fu et al. [188] prepared a biomimetic mussel epoxy resin with cashew phenols and catechol as the main chains through Mannich reaction, and added decyl diamine to mimic protein amino acids (Figure 6a). After curing in seawater, the adhesive obtained has a seawater bonding strength of 17.65 MPa, a tensile strength of 30.31 MPa, and an apparent activation energy of 45.22 kJ mol^−1^. It combines toughening and high adhesion, and the ions in seawater participate in the formation of non-covalent bonds. Li et al. [189] prepared a biomimetic catechol based Mannich base curing agent (CMB) using a one pot condensation reaction (Figure 6b). After bonding with hydrogenated bisphenol A epoxy, the adhesive strength still reached 9.0 MPa even at 4 °C underwater environment. After sandblasting the aluminum surface and curing, the dry bonding strength reached 20.4 MPa, reaching the upper limit of surface bonding after siloxane treatment, and the catechol group had good durability under hydrothermal conditions. Ahn et al. [190] were inspired by mussels to develop a low molecular weight zwitterionic surface wet adhesive (Figure 6c), which has an adhesion energy of approximately 50 mJ /m^−2^ and can self-assemble into a strong adhesive layer with a thickness of less than 4 nm, suitable for nanoprocessing. Zhu et al. [191] developed a biomimetic underwater adhesive (BMUA) containing DOPA, methacrylic acid, N-isopropylacrylamide, and methyl methacrylate (Figure 6d), which triggers hydrogen bonding and hydrophobic crosslinking through solvent exchange. The adhesive strength is greater than 1.4 MPa on various substrates under flowing water (still effective after 45 days), and it has good biocompatibility and hemostatic sealing performance. Liu et al. [192] developed a catalyst free and environmentally friendly silicone adhesive inspired by mussels and applied it to underwater sealing (Figure 6e). It has a shear strength of 0.62 MPa for aluminum overlap (2.1 times higher than traditional silicone rubber), a tensile strength of 0.72 MPa, and can achieve extremely fast underwater curing in the presence of hydrogen peroxide, effectively blocking leaks.

Sandcastle worms secrete adhesive to bond sand particles and shell fragments, providing inspiration for resisting harsh marine environments. Lin et al. [193] developed a biomimetic multifunctional carbon fiber composite material inspired by the sandcastle worm for anti-microbial contamination and fire prevention (Figure 6f). Design an adhesive polymer interface containing casein and ammonium phosphate motifs. The phenolic hydroxyl group of casein enhances the chemical affinity between ammonium polyphosphate, vinyl ester resin, and carbon fiber, while endowing the material with excellent broad-spectrum antibacterial and anti-fouling properties. In conjunction with ammonium phosphate, it achieves ultra-high fire safety levels and is suitable for surface protection of marine facilities. Xu et al. [194] simulated the metal ion assisted coagulation mechanism of sandcastle worm gum and synthesized copolymers containing hydrophobic, bidentate anions, and thermosensitive segments (Figure 6g). Metal ions such as Ca^2+^/Mg^2+^/Cu^2+^ aggregate in solution and undergo temperature enhanced dehydration to prepare adhesives. Its wet bonding strength can be adjusted by ion type, concentration, and coordination time, and can firmly bond glass, silicon, polytetrafluoroethylene, and animal bones. It can also seal leaking pipelines underwater. Rattan binding protein has strong adsorption and corrosion resistance properties. Zhang et al. [195] were inspired by the network structure of rattan binding proteins and synthesized a polyurethane urea adhesive (PUU-FPD) containing large volume hydrophobic “tentacles” and hydrophilic “anchor points”. Hydrophobic side chains remove interface bound water through self-assembly, while hydrophilic groups provide abundant hydrogen bond bridges, which can be applied in various substrates such as PMMA, ceramics, steel, glass, etc. With PTFE, instant adhesion is achieved on wood. After 24 h, the maximum strength reached 1.62 MPa, and it remained stable under conditions such as −196 °C and underwater, and could be released and re bonded multiple times. In the corrosion resistance experiments of metals with different soaking times, the effect was good (Figure 6i). Bui et al. [196] extracted recombinant proteins from barnacles adhesive. It can effectively protect low-carbon steel from corrosion under simulated high salt conditions in the ocean (Figure 6h). This protein interacts with iron ions released from steel substrates through strong surface adsorption, forming a stable protective layer to increase coating impedance and delay corrosion processes, providing molecular guidance for the development of green corrosion inhibitors.

The application comparison of biomimetic adhesives in industrial facilities and marine restoration fields is shown in Table 5. The research on biomimetic underwater adhesives inspired by marine organisms mainly focuses on three types of models: mussels, sandcastle worms, and barnacles. The biomimetic strategy of mussels uses catechol groups as the core bonding unit, and introduces catechol structures through Mannich reaction or copolymerization, which can achieve high-strength bonding in seawater and maintain excellent performance at low temperatures. After long-term service, the bonding attenuation is minimal, and some silicone systems also exhibit good bonding strength, thanks to the efficient displacement of interfacial moisture by catechol [197]. The biomimetic sandcastle worm utilizes coagulation and metal ion coordination to provide adjustable adhesive strength, while also possessing both antibacterial and fire-resistant functions. Barnacle bionics focuses on the collaborative design of hydrophobic “tentacles” and hydrophilic “anchor points”, achieving stable adhesion in extreme temperature, strong acid-base, and high salt environments, and supporting repeated debonding and re sticking. All three strategies start with molecular structure optimization, innovative solidification mechanisms, and interface regulation, effectively overcoming bottlenecks such as slow underwater solidification and weak adhesion. This provides a diverse range of scalable solutions for sealing, anti-corrosion, and emergency maintenance in marine engineering, fully demonstrating the broad prospects of bionics in solving practical underwater bonding problems [198].

### 5.3. Biomedical Sciences

**Figure 7:**
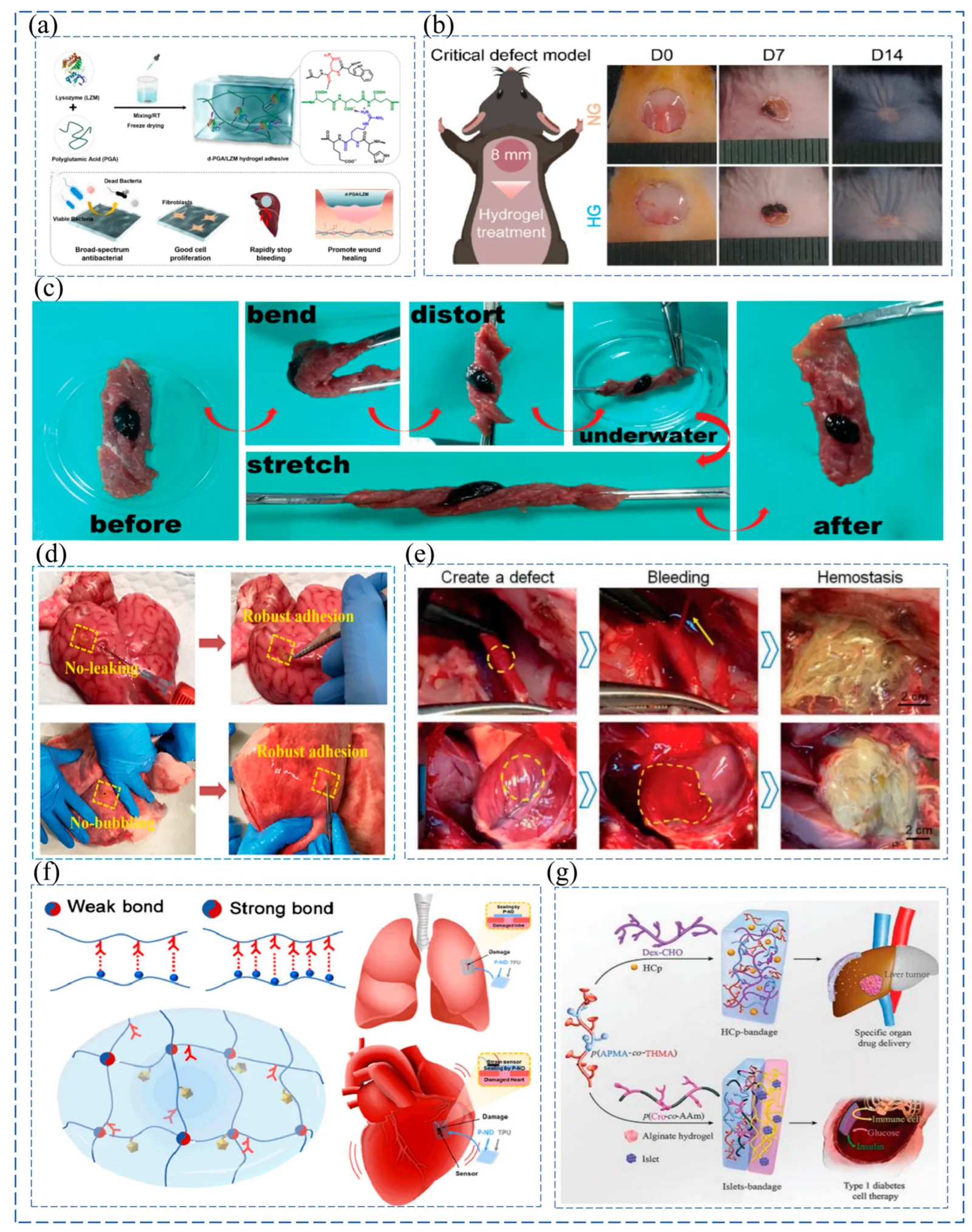
Applications of biomimetic adhesives in biomedicine. (**a**) Sandcastle-worm-inspired polyelectrolyte self-aggregating adhesive composed of poly-γ-glutamic acid and lysozyme [173]. It exhibits strong adhesion and long-term wet stability both underwater and on soft tissue surfaces. Its hemostatic effect is superior to commercial fibrin glue, and the intrinsic antibacterial activity of lysozyme enables efficient antibacterial and wound-healing performance. (**b**) Dual-bionic oxygen-releasing hydrogel inspired by mussel and barnacle cement proteins for wound healing [203]. CaO_2_-mediated oxygen generation and in situ catechol oxidation create a transient high-oxygen microenvironment in the wound area, promoting angiogenesis, red blood cell aggregation, and re-epithelialization. Digital images show the critical defect model at days 0, 7, and 14. (**c**) Mussel-inspired hydrogel adhering to the surface of pig muscle after bending, stretching, distortion, or underwater treatment [200]. The before/after images demonstrate robust, deformable, and water-resistant wet-tissue adhesion. (**d**) Images showing a mussel-inspired biomimetic hydrogel bioadhesive sealing damaged lamb brain tissue and a damaged lamb lung lobe in air and underwater [202]. Hemostatic and sealing performance on heart/lung tissues. The images show no leaking and robust adhesion to blood-covered, dynamic tissue surfaces. (**e**) Macroscopic images of the hemostatic effects of a dual-bioinspired hydrogel, inspired by mussels and barnacles, on bleeding from puncture wounds in a pig’s femoral artery and heart [201]. The adhesive rapidly stops bleeding and seals the tissue, demonstrating emergency hemostatic capability. (**f**) A polyelectrolyte hydrogel inspired by the sandcastle worm acts as a bioadhesive for various tissues, promoting wound healing and suppressing inflammation [199]. Schematic of weak-bond/strong-bond networks and application to lung/heart injury. The load-sharing effect of multiple hydrogen-bond clusters provides strong adhesion to glass, plastic, and biological tissue without relying on chemical reactions. (**g**) A mussel-inspired adhesive hydrogel with a “load-sharing” mechanism applied to a tissue bandage for drug and cell delivery [204]. Tissue adhesive bandage for drug and cell delivery. The system involves HCP-banding, P(AM/APMA-co-TRISMA), p(γ-GA), alginate hydrogel, and islets. It enables specific organ drug delivery for liver cancer and islet anchoring for type 1 diabetes blood glucose control. Arrows indicate delivery, anchoring, and therapeutic action.

Severe bleeding and wound infection caused by trauma are the two main causes of death and disability in trauma emergency care. Traditional suturing and gauze have weak adhesion to wet and dynamic tissue surfaces, delayed hemostasis, and are difficult to balance antibacterial and healing functions. In recent years, bionics has injected new vitality into the design of bio adhesives: sandcastle worms rely on polyelectrolyte condensation to achieve underwater fixation, mussels form strong wet adhesion with catechol groups, barnacles remove interfacial water through hydrophobic drainage, and enhance interfacial interlocking through nanoscale molecular permeation. These strategies are used to build hydrogel systems, respectively or cooperatively, while integrating active functions such as oxygen production, antibacterial, drug delivery, etc. Liu et al. [173] were inspired by the electrostatic complexation mechanism of cations and anions in the protein of Sandcastle worm, and developed a polyelectrolyte self-aggregating adhesive of poly-γ-glutamic acid (PGA) and lysozyme (LZM) (Figure 7a). The adhesion force of this system to solid plates underwater exceeds 250 kPa, and to soft tissues exceeds 50 kPa. After soaking for 7 days, it still maintains 80 kPa, demonstrating rare long-term wet stability. Its hemostatic effect is superior to commercial fibrin glue in rat liver and tail defect models. The inherent antibacterial activity of lysozyme endows it with nearly 100% inhibition rate against Staphylococcus aureus and Escherichia coli, and the healing rate of infected wounds is about 100% within 15 days. Cong et al. [199] synthesized polyampholyte hydrogel (Figure 7f) inspired by the sandcastle worm, which uses ionic bonds as physical crosslinking points to endow the material with excellent mechanical properties. At the same time, the rich carboxyl groups provided by acrylic acid can instantly form hydrogen bonds on the surface of wet tissue and interact with static electricity to achieve rapid adhesion. The gel showed a firm fit on a variety of rat organs, and had good biocompatibility in vitro and in vivo, which could significantly inhibit inflammation and accelerate the re epithelialization of the wound surface.

Yang et al. [200] fused the drainage adhesion of mussels, the molecular penetration of ivy and the enhanced cross-linking mechanism of oysters into the collagen starch (CoSt) hydrogel and applied it in the biomedical field. This material can quickly remove interfacial water molecules, enhance energy dissipation, and introduce multiple reversible dynamic bonds. Its wet adhesion force reaches 62 ± 4.8 kPa, sealing pressure is as high as 153.2 ± 35.1 mmHg (far exceeding physiological systolic pressure), and it has self-healing and injectability. In various models such as rat tail amputation, skin incision, severe liver injury, abdominal aorta and nerve transection, CoSt demonstrated superior hemostatic effects compared to fibrin glue due to its tough wound closure and red blood cell capture ability (Figure 7c). Inspired by mussel and barnacle cement proteins, Pan et al. [201] constructed a multi network hydrogel, which made use of rich phenolic hydroxyl, tough dissipative matrix and phenol quinone dynamic redox balance to obtain 151.40 ± 1.50 kPa of repeatable wet adhesion, with rapid self-healing and broad-spectrum antibacterial properties. In the pig heart perforation and femoral artery injury models (Figure 7e), its hemostatic efficiency significantly exceeded that of commercially available gauze, and when combined with commercial hemostatic sponges, it showed great potential for deep combat injuries. Wu et al. [202] put forward PTLA hydrogel composed of tannic acid, hyperbranched polylysine and acrylic acid for extreme environment. With 3D printing, personalized customization can be achieved, and ultra-fast sealing can be completed within 5 s. The excellent hemostatic effect was validated in vitro lamb and in vivo rat models (Figure 7d). In addition, the material is frost resistant, pressure resistant, and has a self-consolidating dry powder form, making it easy to store and carry on the battlefield for a long time.

Lee et al. [203] were inspired by mussels and introduced CaO mediated oxygen generation crosslinking reactions to develop gelatin based oxygen producing tissue adhesives. The adhesive force of the hydrogel is 15–38 kPa, but it can rapidly release up to 70% pO_2_ of oxygen, and build a temporary hyperoxic microenvironment on the local part of the wound, thus significantly promoting angiogenesis, red blood cell aggregation and re epithelialization. In three mouse experiments set at different times, the production of oxygen hydrogel by bisimilation accelerated skin healing (Figure 7b). This is due to the coupling of oxygen generation function with DA polymerization and cross-linking to realize the synchronization of gel and oxygen release. Chen et al. [204] introduced the “triple hydrogen bond cluster” (THBC) as a side group into the mussel biomimetic hydrogel (Figure 7g), and relied on the “load sharing” effect to achieve strong adhesion to glass, plastic, PTFE and even biological tissues without relying on chemical reactions, and based on this, made tissue adhesive bandages. This bandage can be used for targeted sustained release of nanomedicine for local chemotherapy of liver cancer, achieving almost complete tumor inhibition in a mouse in situ liver cancer model; it can also encapsulate islet cells, anchor them in vivo, and maintain normal blood sugar in chemically induced diabetes mice for about 1 month.

Severe bleeding and wound infection caused by trauma are the main causes of death and disability in trauma emergency care. Traditional suturing and gauze have weak adhesion to wet and dynamic tissue surfaces, delayed hemostasis, and are difficult to balance antibacterial and healing functions. Inspired by biomimetic strategies such as polyelectrolyte condensation of the Shabu worm, wet adhesion of mussel catechol, hydrophobic drainage of barnacles and nano osmotic interlocking, researchers constructed a variety of hydrogel adhesives and integrated functions such as oxygen production, antibacterial and drug delivery. The self-aggregating adhesive of poly (γ- glutamic acid) and lysozyme exhibits strong adhesion and long-term wet stability both underwater and on soft tissue surfaces. Its hemostatic effect is superior to commercial fibrin glue, and it utilizes the inherent antibacterial activity of lysozyme to achieve efficient antibacterial and wound healing. The polyampholyte hydrogel can quickly form hydrogen bond and electrostatic interaction on the surface of wet tissue with ionic bond physical cross-linking and rich carboxyl groups, which can achieve a firm fit, good biocompatibility, inhibit inflammation and accelerate re epithelization. Collagen starch hydrogel integrates drainage adhesion, molecular penetration and strengthening cross-linking mechanism, which can quickly remove interface water, enhance energy dissipation and introduce multiple reversible dynamic bonds. It has self-healing and injectability, and its hemostatic effect is better than that of fibrin glue in a variety of bleeding and tissue damage models. With the help of phenol hydroxyl, tough dissipative matrix and phenol quinone dynamic oxidation-reduction balance, multi network hydrogel can obtain repeatable wet adhesion, which has both rapid self-healing and broad-spectrum antibacterial properties. Its hemostatic efficiency in heart and artery injuries exceeds that of commercial gauze, and its combination with hemostatic sponge has potential for deep war trauma. The hydrogel composed of tannic acid, hyperbranched polylysine and acrylic acid can be customized through printing to achieve ultrafast sealing, and has the form of antifreeze, pressure resistance and self-gelling dry powder, which is convenient for long-term storage and battlefield carrying; Gelatin based oxygen producing tissue adhesive crosslinks through calcium oxide mediated oxygen generation, creating a brief high oxygen microenvironment in the wound area, promoting angiogenesis, red blood cell aggregation, and re epithelialization, and accelerating skin healing. The mussel biomimetic hydrogel with multiple hydrogen bond clusters relies on the load sharing effect to strongly adhere to glass, plastic and even biological tissue without relying on chemical reaction. The tissue adhesive bandage can be used for the targeting and continuous release of local chemotherapy nano drugs for liver cancer and significantly inhibit tumor growth, and can also encapsulate islet cells and anchor them in vivo to maintain normal blood sugar in diabetes models. Overall, these biomimetic adhesives have achieved multifunctional integration through multi mechanism synergy, including strong adhesion between wet and underwater environments, rapid hemostasis, antibacterial properties, healing promotion, and drug and cell delivery. They have shown superior application prospects compared to traditional materials in various animal models and extreme environments.

The application comparison of biomimetic adhesives in industrial facilities and marine restoration fields is shown in Table 6. These systems can generally form strong interfacial bonding in wet or underwater environments, and some materials also exhibit long-term stability, reusability, and adaptability to multiple substrates, demonstrating the potential of biomimetic adhesion strategies in complex interface repair. However, the testing conditions mentioned in this table are mostly focused on biomedical scenarios and cannot be directly equivalent to the actual working conditions in industrial facilities and marine remediation. Industrial and marine environments often involve surfaces of metals, concrete, and composite materials, as well as factors such as salt spray, microbial fouling, and water erosion. Therefore, existing results suggest that biomimetic adhesives have the potential to expand into these fields, rather than having been fully validated. In the future, if it is aimed at industrial facilities and marine restoration, it is necessary to systematically evaluate their adhesion durability, sealing reliability, environmental adaptability, and feasibility of large-scale construction in real or simulated engineering environments.

### 5.4. Summary

The overall application comparison of biomimetic adhesives is shown in Table 7. Although significant breakthroughs have been made in wet adhesion strength, hemostatic speed, and antibacterial function in current research on biomimetic adhesives, their clinical translation still faces multiple systemic limitations. Most wet adhesion tests use fresh ex vivo tissue or flat surfaces, while the actual wound is continuously washed away by blood and tissue fluid, accompanied by dynamic deformation caused by cardiac pulsation or respiratory movement. The reported adhesion data under these complex conditions may be overestimated, lacking systematic tracking of adhesion stability and mechanical performance degradation over several months or even years [197]. At the antibacterial level, in vitro inhibition zones or co culture data are difficult to simulate the competitive environment of multiple microorganisms in vivo, and most studies have not evaluated the potential interference of long-term antibacterial agent release on normal microbial communities or tissue regeneration. A large number of hemostasis experiments are limited to acute models such as tail amputation and liver puncture in mice, and there is a significant difference between them and the real scenarios of high blood pressure spraying or deep penetrating injuries in human arteries. Moreover, the comparison with the clinical gold standard is mostly based on a single indicator, lacking multi center and large sample evidence-based verification [198].

Multi network or dual biomimetic systems, due to their complex components and insufficient controllability of inter batch cross-linking and mechanical properties, make it difficult for 3D printing to meet the immediate needs of emergency situations, despite its personalized precision. The efficacy game between drug/cell delivery function and adhesive sealing is rarely quantitatively balanced. In the future, consensus must be established on complex dynamic wet environment models, standardized hemostatic efficacy evaluation, and multi center animal validation, and mechanism analysis must be deepened in degradation regeneration time matching, multifunctional collaborative windows, and other aspects to truly translate laboratory indicators into clinical benefits [205,206].

## 6. Conclusions and Outlook

From the above three types of biomimetic strategies, it can be seen that the most prominent phenomenon is that the reported performance depends more on the material type, the adhered substrate, and the testing scheme, rather than the biomimetic inspiration itself. On well prepared metal or glass substrates, rigid catechol thermosetting resins and peptide aggregates can reach megapascals. For example, enzyme catalyzed polymerization of DOPA rich peptides can stabilize at 31.8 MPa in the range of −196 to 150 ° C [161], 8.1 MPa on steel under seawater conditions [167], 17.65 MPa after seawater solidification [188], and still reach 9.0 MPa even at low temperatures of 4 ° C [189]. However, the soft hydrogel adhesive on wet tissue is still one to two orders of magnitude lower, usually only 50–250 kPa [173,199,200,201]; the oxygen or drug loading system is further reduced to 15–38 kPa [203]; wearable elastomers are in the middle range of about 1.5–2.8 MPa [179,180,181,182]. This performance span is often described as ‘steady progress’, but there is currently no unified system that is firmly adhered to wet tissues, resistant to cyclic loads, scalable, and biocompatible. It needs to be critically pointed out that many ‘headline data’ are not comparable to each other. The same hydrogel is about 50 kPa under 10 N preload, and 238 kPa under 100 N preload [122]. Overlapping shear, probe adhesion, 90°/180° peel off, and burst pressure tests detect different failure modes with varying dimensions, but they are rarely converted into a unified interfacial fracture energy. The curing history further interferes with comparability: the joint cured underwater for 72 h cannot be equivalent to the sample tested immediately after pressurization [122]. Almost all data is taken from smooth, clean, and even substrates such as glass, steel, and pigskin, rather than rough, soiled, greased, or dynamically moving surfaces. The failure modes closely related to long-term durability (adhesive failure compared to cohesive failure) and crack propagation resistance are rarely reported together with peak strength. The result is that the ‘strongest’ adhesive is often measured under the most favorable and unrealistic conditions, rather than the one that performs best in actual service.

The gap between laboratory indicators and service conditions is the second and more serious limitation. Industrial and marine bonding requires maintenance for several years under salt spray aging, microbial fouling, hydraulic shear, and temperature cycling. However, the longest durability reports in this field are still only 45 days under flowing water [191] and 50 days under static immersion [119]; there is a basic lack of systematic data on seawater aging, cathode stripping, and freeze-thaw fatigue. In the biomedical field, the most convincing evidence of hemostasis still comes from mouse tail amputation and liver puncture models. This cannot reproduce the situations of high pressure, pulsation, and deep penetrating bleeding in human trauma, nor can it simulate the competitive multi microbial wound environment in the body. Biocompatibility evaluation is usually limited to acute cytotoxicity and short-term skin contact, lacking evidence of chronic inflammation, degradation products, and organ level. For wearable devices, the attenuation of adhesion and conductivity due to swelling caused by sweat or long-term immersion, as well as the hysteresis and cyclic fatigue of thermally reversible adhesive bonds, have not been quantified. The above-mentioned deficiencies in measurement and transformation are compounded by a design logic that still relies on experience. Although the same batch of literature has shown that these variables constrain each other: increasing catechol content can increase interfacial energy, but it can accelerate the cohesive loss caused by oxidation. Increasing crosslinking density can enhance the body, but it will inhibit chain entanglement and wetting spreading. Integrating oxygen, drug, or conductive functions into an adhesive matrix can lower the adhesion of wet tissue from greater than 250 kPa to 15–38 kPa [173,199,200,201,202,203]. Therefore, multifunctionality is often achieved at the cost of sacrificing the adhesion performance it serves, and the question of “to what extent additional functionality becomes a net burden” has not yet been systematically characterized. Based on these observations, the primary shift in this field is from pursuing peak intensity to standardized evaluation that matches the application environment: establishing a unified set of substrates (covering rough, grease covered, and soiled surfaces), fixing preload and contact time, mandatory reporting of failure modes and interfacial fracture energy, and directly comparing with clinical gold standards such as cyanoacrylate, fibrin glue, and sutures on the same animal model. The design energy should be focused on the weakest links rather than the most dazzling functions, namely long-term stability under dynamic loads, degradation cycles that match tissue remodeling, and scalable aqueous phase manufacturing processes. With the help of in situ spectroscopy, liquid-phase electron microscopy, and molecular dynamics simulations, the nanoscale water film displacement process should be truly linked to the macroscopic bonding life. The data-driven approach should not only be used to pursue higher strength, but also applied to quantitatively characterize the trade-off surface between adhesion, cohesion, and additional functionality, thereby accelerating the discovery of non-DOPA based adhesion elements. Only when adhesives are compared horizontally on real, standardized indicators and optimized based on service conditions rather than the number of integrated functions, can these laboratory advances truly become adhesives that can be reliably held underwater, on moist tissues, and on moving skin.

## Figures and Tables

**Table 1:** Comparison of three bionic adhesion mechanisms.

Comparative Dimension	Mussel	Barnacle	Sandcastle Worm
Core mechanism	Chemical adhesion: DOPA mediated metal coordination and crosslinking reactions.	Chemical adhesion: Proteins self-assemble into amyloid fibers, which are enzymatically crosslinked and solidified.	Chemical and physical synergy: charged proteins undergo liquid–liquid phase separation to form a condensed layer, followed by ion crosslinking and solidification.
Key molecule	Mytilus edulis foot protein (Mfp-3/5) is rich in DOPA (catechol groups).	Barnacle cement proteins (containing cysteine) forms β—folded amyloid fibers.	Positive and negative charge pairing proteins DOPA, Phosphates and magnesium ions (Mg^2+^).
Adhesion process	• The feet secrete liquid protein; • DOPA coordinates with metal ions (Fe^3+^); • Oxidative crosslinking forms solid adhesive plaques.	• Secretory protein coagulation layer releases interface water; • Self-assemble into an amyloid fiber network; • Enzymatic oxidation cross-linking, permanent solidification.	• Secreting positive and negative electrical proteins, triggering liquid–liquid phase separation in seawater; • Wetting the substrate with a condensed layer and draining the moisture; • pH and Mg^2+^ induce phase transition and solidify into a porous solid.
Structural characteristics	Foot fibers: core (collagen, impact resistant), substrate (adhesion).	Adhesive layer: a high-density, water-insoluble protein network with high hardness.	Tubular nest: Glue binds sand particles/shell fragments, repairable.

**Table 2:** Comparison of shear strength and testing conditions of representative biomimetic wet adhesive.

System	Adhesive Strength	Test Conditions	Ref.
Biomimetic sandcastle worm	498.42 kPa (underwater curing for 72 h); 451.95 kPa (after 50 days of immersion)	Underwater solidification, overlapping shear, 50 day soaking	[119]
Imitation barnacles/articular cartilage amphibious adhesive (P3/SiW condensate)	Underwater lap shear 4.2 MPa	Underwater	[120]
Bionic sandcastle worm (PAMP hydrogel)	The underwater bonding strength reaches 56.9 kPa, and the debonding work is 45.1 J/m^2^	Standardized lap shear, 180° peel off	[121]
Mussel (silicone adhesive)	Tape press back: 238 kPa	Underwater	[122]

**Table 3:** Comparison of synthesis routes for biomimetic wet-state adhesives.

Comparative Dimension	Mussel	Barnacle	Sandcastle Worm
Key functional groups and molecular structure	• Core group: DOPA (catechol) • Synergistic groups: Lysine/Arginine (cation), Fe^3+^ coordination site • Typical proteins: Mfp-3/5 (high DOPA content), Mfp-6 (containing cysteine, providing reducing protection)	• Core structure: hydrophobic/hydrophilic block copolymer, rich in cysteine (disulfide bond) • Interface proteins: cp19k (block copolymer-like sequence), cp20k (rich in Cys) • Ontology protein: cp52k/cp100k (forming amyloid-like fibers)	• Negative charged group: phosphorylated serine (phosphate group, strong metal coordination) • Positive electrode group: cationic protein rich in glycine/lysine (Pc1-5) • Crosslinking assistance: DOPA, Mg^2+^/Ca^2+^
Main synthetic pathways and methods	Path 1 (peptide synthesis): Solid-phase peptide synthesis (SPPS), recombinant protein expression, NCA ring opening polymerization, two-step enzyme catalysis (tyrosinase mediated hydroxylation); Path 2 (Modified Polymer): Grafting or copolymerizing catechol groups onto polyurethane/epoxy/polysaccharide skeleton.	Path 1 (condensed layer adhesive): hydrophobic modification of end groups (such as PPG grafted butyl), spontaneous phase separation upon contact with water; Path 2(copolymerization simulation): Cationic monomer (ATAC) and aromatic monomer (PEA) are copolymerized to construct a π–π/cation–π network.	Path 1 (peptide solid-phase synthesis): Efficient synthesis of specific sequence anionic/cationic peptides (purity > 95%); Path 2 (NCA ring opening polymerization): Synthesize copolymer peptides with adjustable phosphorylation degree and DOPA content; Path 3 (Functional Molecular Grafting): Grafting catechol/phosphate groups onto synthetic frameworks such as epoxybutane.

**Table 4:** Comparison of application of biomimetic adhesive in wearable device field.

Representative System	Indicator Type	Value	Test Conditions	Contact/Curing Condition	Substrate	Ref.
Biomimetic Mussel (PLA-LM 3D bead-like structure)	Lap shear	2.7625 MPa; +10.77% after solar irradiation	Substrate, wet/dry state	/	/	[179]
Biomimetic Mussel (Anti-sweat bioelectronic skin)	Ionic conductivity	1.52 S/m and −40 °C freeze resistance	Stretched at multiple low-temperature points including −80 °C	/	Skin	[180]
Biomimetic Barnacle (MXene/PHMP hydrogel)	Conductivity, pressure Sensitivity	Conductivity up to 0.016 S/m, pressure sensitivity 0.63 kPa^−1^	On various substrates such as glass, PET, and pigskin	Repeatable wet adhesion	Glass, PET, porcine skin	[181]
Biomimetic Mussel (Hydrogel-plastic hybrid)	Adhesive strength/Interfacial toughness	1.52 MPa; 1450 J/m^2^	Temperature response; Human skin 10 min	Switching ratio > 1000	Plastic/hydrogel	[182]
Biomimetic Barnacle (CP hydrogel)	Adhesion strength	22.8 kPa	Stretchability 1430%, conductivity 18.5 mS/cm	Cation–π, hydrophobic, ionic-bond coordination	Human skin	[184]
Mussel and Barnacle dual-inspired (AHBH bilayer hydrogel)	Peel force	59.7 N/m	Hydrophobic layer 0.5 h, contact angle 133.87°	Contact angle 133.87°; 8 s self-crosslinking	Skin/electrode	[185]
Biomimetic Sandcastle Worm (Xerogel)	Adhesion energy	>350 J/m^2^	8 s self-crosslinking; conductivity >2.5 S/m	Recyclable and reconfigurable	/	[186]

**Table 5:** Comparison of application of biomimetic adhesives in industrial facilities and marine restoration fields.

Representative System	Indicator Type	Value	Test Conditions	Substrate	Curing/Contact Condition	Ref.
Biomimetic mussel	Bonding strength	17.65 MPa	Sewage curing, seawater	Steel, etc.	Seawater curing	[188]
Biomimetic mussel (CMB curing agent)	Adhesive strength	4 °C underwater 9.0 MPa; Dry state 20.4 MPa	Sandblasted aluminum; Hydrothermal durability	Sandblasted aluminum	Hydrothermal durability	[189]
Biomimetic mussel (Zwitterionic wet adhesive)	Adhesion energy	Approx. 50 mJ/m^2^	Self-assembly less than 4 nm	/	Self-assembled layer < 4 nm	[190]
Biomimetic mussel	Adhesion strength	1.4 MPa, effective for 45 days	Under flowing water, multiple substrates	Multiple substrates, aluminum	H_2_O_2_ underwater curing; bulk tensile 0.72 MPa	[191]
Biomimetic mussel	Lap shear	0.62 MPa	Aluminum; H_2_O_2_ underwater curing; Bulk tensile 0.72 MPa	Aluminum, etc.	Catalyst-free; H_2_O_2_ curing	[192]
Biomimetic sandcastle worm (PUU-FPD)	Adhesion strength	24 h maximum 1.62 MPa	Multiple substrates; −196 °C/underwater	PMMA, ceramics, steel, glass, PTFE, wood	24 h; repeatable debonding/rebonding	[195]
Biomimetic barnacle	Corrosion resistance	24-h immersion, approx. 2% corrosion on steel surface	Steel at 25 °C	Low-carbon steel	24 h	[196]

**Table 6:** Comparison of biomedical applications.

Representative System	Indicator Type	Value	Test Conditions	Ref.
Biomimetic sandcastle Worm (PGA-LZM coacervate)	Adhesion force (Mode unspecified)	Solid plate > 250 kPa; Soft tissue > 50 kPa; 7 days 80 kPa	Underwater, 7-day immersion	[173]
Biomimetic mussel (CoSt hydrogel)	Wet adhesion force/sealing pressure	62 ± 4.8 kPa; 153.2 ± 35.1 mmHg	Various rat hemorrhage models	[200]
Barnacle (Multi-network hydrogel)	Repeatable wet adhesion	151.40 ± 1.50 kPa	Porcine heart, femoral artery injury	[201]
Biomimetic mussel (Oxygen-generating gelatin)	Adhesion force	15–38 kPa	Mouse skin healing; 70% pO2	[203]
Biomimetic mussel (THBC bandage)	Strong adhesion	/	Glass/Plastic/PTFE/Tissue	[204]

**Table 7:** Comparison of applications for biomimetic adhesives.

Comparative Dimension	Biomimetic Strategy	System	Core Performance	Limitation	Test Conditions
Wearable devices	Mussels (DOPA/cationic–π), barnacles (hydrophobic drainage), sandcastle worms (coagulation/ion crosslinking).	PLA-LM 3D structure, anti-sweat bio electronic skin, MXene/PHMP hydrogel, CP hydrogel, PTA printing adhesive, etc.	High stretchability, self-healing, freeze resistance, underwater adhesion, conductivity, etc.	Long term immersion swelling, cyclic performance degradation, and insufficient biosafety verification (mostly short-term).	Wet/dynamic physiological environment; underwater/air; −40/−80 °C; human skin, glass, PET, porcine skin; cyclic loading/solar irradiation
Industry and ocean restoration	Mussels (catechol/metal coordination), barnacles (hydrophobic hydrophilic synergy), sandcastle worms (coagulation/ion crosslinking).	Catechol epoxy resin, CMB curing agent, PUU-FPA adhesive BMUA, Silicone sealant, carbon fiber composite materials, etc.	The bonding strength of seawater reaches 17.65 MPa, low-temperature curing (4 °C), corrosion resistance, fire resistance, repeatable bonding, and extreme temperature resistance.	Large scale construction is difficult, and there is insufficient long-term data on seawater aging.	Seawater/flowing water/underwater; 4 °C; −196 to 150 °C; sandblasted aluminum/steel/PMMA/ceramics/glass/PTFE/wood; 24 h/45 days; high salinity
Biomedicine	Sandburg worm (polyelectrolyte coagulation), mussels (DOPA/hydrogen bonding), barnacles (hydrophobic drainage/protein cross-linking).	PGA-LZM condensate, CoSt hydrogel, PTLA 3D printing glue, double bionic water gel, THBC bandage, oxygen generating gelatin adhesive, etc.	Wet adhesion force > 50 kPa, better hemostatic efficiency than fibrin glue, antibacterial (>99%), promoting healing, controllable drug/cell release.	Complex components and poor batch stability.	Underwater/soft tissue; porcine skin/heart/artery/liver; diabetic/infected wound models; rat/mouse; in vivo; dynamic wet tissue

## Data Availability

No new data were created or analyzed in this study. Data sharing is not applicable to this article.
